# Cocaine-Induced DNA-Dependent Protein Kinase Relieves RNAP II Pausing by Promoting TRIM28 Phosphorylation and RNAP II Hyperphosphorylation to Enhance HIV Transcription

**DOI:** 10.3390/cells13231950

**Published:** 2024-11-23

**Authors:** Adhikarimayum Lakhikumar Sharma, Priya Tyagi, Meenata Khumallambam, Mudit Tyagi

**Affiliations:** Center for Translational Medicine, Thomas Jefferson University, 1020 Locust Street, Philadelphia, PA 19107, USA; lakhikumarsharma.adhikarimayum@jefferson.edu (A.L.S.); ptyagi28@terpmail.umd.edu (P.T.); mxk848@jefferson.edu (M.K.)

**Keywords:** cocaine, DNA-PK, HIV transcription, HIV gene expression, replication, RNA polymerase, TRIM28, RNAP II pause release, elongation

## Abstract

Drug abuse continues to pose a significant challenge in HIV control efforts. In our investigation, we discovered that cocaine not only upregulates the expression of the DNA-dependent protein kinase (DNA-PK) but also augments DNA-PK activation by enhancing its phosphorylation at S2056. Moreover, DNA-PK phosphorylation triggers the higher localization of the DNA-PK into the nucleus. The finding that cocaine increases the nuclear localization of the DNA-PK provides further support to our observation of enhanced DNA-PK recruitment at the HIV long terminal repeat (LTR) following cocaine exposure. By activating and facilitating the nuclear localization of the DNA-PK, cocaine effectively orchestrates multiple stages of HIV transcription, thereby promoting HIV replication. Additionally, our study demonstrates that the cocaine-induced DNA-PK promotes the hyper-phosphorylation of the RNA polymerase II (RNAP II) carboxyl-terminal domain (CTD) at Ser5 and Ser2 sites, enhancing both the initiation and elongation phases, respectively, of HIV transcription. The cocaine-mediated enhancement of transcriptional initiation is supported by its activation of cyclin-dependent kinase 7 (CDK7). Additionally, the induction of transcriptional elongation is marked by higher LTR recruitment and the increased phosphorylation of CDK9, which indicates the stimulation of positive transcriptional elongation factor b (P-TEFb). We demonstrate for the first time that cocaine, through DNA-PK activation, promotes the specific phosphorylation of TRIM28 at serine 824 (p-TRIM28, S824). This modification converts TRIM28 from a transcriptional inhibitor to a transactivator for HIV transcription. Additionally, we observed that the phosphorylation of TRIM28 (p-TRIM28, S824) promotes the transition from the pausing phase to the elongation phase of HIV transcription, thereby facilitating the production of full-length HIV genomic transcripts. This finding corroborates the previously observed enhanced RNAP II CTD phosphorylation at Ser2, a marker of transcriptional elongation, following cocaine exposure. Accordingly, upon cocaine treatment, we observed the elevated recruitment of p-TRIM28-(S824) at the HIV LTR. Overall, our results unravel the intricate molecular mechanisms underlying cocaine-induced HIV transcription and gene expression. These findings hold promise for the development of highly targeted therapeutics aimed at mitigating the detrimental effects of cocaine in individuals living with HIV.

## 1. Introduction

The onset of acquired immunodeficiency syndrome (AIDS), triggered by Human Immunodeficiency Virus type 1 (HIV), is one of the most profoundly impactful diseases humanity has faced. Since the identification of HIV in 1981, extensive endeavors have been undertaken to combat HIV infection. These efforts have catalyzed significant progress in the realms of immunology and HIV virology, marking notable advancements along the way [1,2,3,4]. However, HIV eradication or a preventive vaccine is yet to be developed [5]. The current anti-HIV drug regimens (anti-retroviral therapy, ART) have been highly successful in lowering HIV/AIDS-related mortality and improving the quality of life for people living with HIV (PLWH) [1,6]. As ART can effectively diminish the viral load to undetectable levels through standard methodologies, the substantially decreased levels of HIV while receiving ART facilitates the restoration and sustenance of a robust immune system. This restoration enables the body to effectively defend against opportunistic infections and illnesses [7,8,9]. In addition, ART greatly reduces the risk of HIV transmission [7]. On the other hand, dangerous behavior, such as unprotected sex and needle sharing by illicit drug users, significantly increases HIV transmission risk [10,11]. Although there have been remarkable achievements in controlling HIV, the prevalence of illicit drug usage remains a significant contributor to new HIV infection due to drug users’ perilous behavior [12,13,14,15,16,17]. Cocaine (Coc), a powerfully addictive stimulant drug, has a high potential for abuse tendency [18,19,20,21]. Cocaine is primarily used orally, intranasally, intravenously, or by inhalation [22]. Continuous use of cocaine interferes with normal brain function; thus, it compromises judgment and decision-making capabilities, leading to risky behavior such as needle sharing and sexual behavior, including trading sex for drugs [23,24]. Once infected, cocaine further increases the severity of the HIV infection; stimulates HIV replication, including in the central nervous system (CNS); and accelerates the occurrence of neurocognitive impairments [25,26,27,28]. Studies have also documented that cocaine use accelerates CD4+ T cell loss, even in ART-treated individuals [29,30]. However, the precise mechanisms by which cocaine and HIV synergize to compromise the health of individuals living with HIV (PLWH) remain unclear.

Similar to host cell gene transcription, RNA polymerase II (RNAP II) is required for HIV transcription. RNAP II is regulated by specific phosphorylation events in the carboxyl-terminal domain (CTD) of the RNAP II large subunit [31]. The human RNAP II CTD consists of 52 tandem repeats of a consensus sequence Tyr1-Ser2-Pro3-Thr4-Ser5-Pro6-Ser7 [32,33,34,35]. Many known kinases can phosphorylate the RNAP II CTD. However, the most notable kinases that phosphorylate RNAP II are cyclin-dependent kinase 7 (CDK7), which phosphorylates RNAP II at Ser5, and CDK9, which phosphorylates RNAP II at Ser2 [31,34,36]. Our previous studies documented that the DNA-PK can phosphorylate RNAP II CTD in all three serine residues (Ser2, Ser5, and Ser7) [37]. We have also shown that the transactivator of the transcription (Tat) protein, which is vital for HIV transcription, is a potential substrate of the DNA-PK [37]. Data generated from our previous study also suggested that cellular activation augments both the nuclear translocation and HIV LTR recruitment of the DNA-PK [37,38,39].

The DNA-PK, a protein kinase, requires association with DNA to become catalytically active. The DNA-PK holoenzyme consists of two components: a 450 kDa catalytic subunit (DNA-PKcs), which is a serine/threonine kinase, and a regulatory component known as Ku, a heterodimer of Ku70 and Ku80 [40,41,42]. The DNA-PK is well studied for its role in repairing DNA damage and maintaining the stability of the genome, including during V(D)J recombination [43,44,45]. The DNA-PK especially plays a crucial role in the non-homologous end-joining (NHEJ) DNA repair pathway [46]. While multiple recent studies, including our own, have suggested the potential involvement of the DNA-PK in transcriptional regulation [37,47], the precise role of the DNA-PK in the transcription process was delineated by our research [37]. It has been documented that the DNA-PK interacts with various transcription factors and components of the transcription machinery [47]. Notably, the DNA-PK not only engages with numerous transcription factors, such as TFIIH, P-TEFb, p53, NF-κB, and SP1, but also modulates their activity through phosphorylation. These interactions typically amplify the expression of genes regulated by these transcription factors.

During HIV transcription, the phosphorylation of the RNAP II CTD at the position Ser5 is associated with the early stages of transcription, particularly transcription initiation. This modification recruits capping enzyme complexes that add a 7-methylguanosine cap to the nascent RNA molecule, which protects the RNA from degradation and later facilitates its processing. However, the phosphorylation of the RNAP II CTD at Ser2 is linked to the elongation phase of transcription, as this post-translational modification of RNAP II makes it processive or elongation-proficient, as it reduces the slipping of RNAP II from the DNA template. This modification also facilitates the recruitment of transcription factors involved in mRNA maturation and processing, including splicing and polyadenylation. For efficient transcription elongation, not only processive RNAP II is required, but also the removal of negative transcription factors (NFs), which promote promoter-proximal pausing of RNAP II, is essential [48,49,50,51,52]. Analogous to cellular gene expression, HIV transcriptional initiation also halts after generating short nascent mRNA of around 60 nucleotides due to the binding of negative transcription factors (NFs) at the HIV LTR [53,54,55]. Some notable NFs are the negative elongation factor (NELF) and the 5,6-dicholoro-1-β-d-ribofuranoxylbenzimidazole (DRB) sensitivity-inducing factor (DSIF) [56,57]. Recently, in addition to the DSIF and the NELF, another factor, TRIM28 (tripartite motif-containing protein 28), also known as KAP1 (KRAB-associated protein 1) and transcription intermediary factor 1β (TIF1β), has been shown to promote promoter-proximal pausing at cellular gene promoters [39,58,59,60]. TRIM28 was originally identified as a transcriptional corepressor due to its interaction with the members of the Kruppel associated box (KRAB) which contains the transcription factor family [61,62]. This factor has since been shown to be a highly versatile multidomain protein, involved in the regulation of numerous genes across the genome, including endogenous retroviruses [58,59,63,64,65]. TRIM28 suppresses transcription by binding to KRAB-containing transcription factors, leading to the recruitment of co-repressors, histone deacetylases (HDACs), and chromatin remodeling complexes. This results in the compaction of the chromatin structure and the inhibition of gene transcription. It has been noted that in many inactivated genes, TRIM28 stabilizes the pausing of RNAP II near the transcriptional start site (TSS), which promotes promoter-proximal pausing and the accumulation of RNAP II near the gene promoter [58]. The modulation of RNAP II pausing depends on the phosphorylation of TRIM28 at the specific site, Ser824. Similar to the SPT5 subunit of the DSIF, the phosphorylation of TRIM28 is crucial in converting it from a pausing or negative elongation factor to a positive elongation factor [39,58,59,66]. The DNA-PK is the principal kinase that directly interacts with TRIM28 and catalyzes the phosphorylation of TRIM28 at serine 824 residue (p-TRIM28, S824), converting it to an elongation factor [39,60]. However, pertaining to HIV transcription, the role of TRIM28 is still not clearly defined. Nevertheless, TRIM28 is shown to play a complex role in controlling the transcription of HIV and other DNA/RNA viruses, influencing both positive and negative regulatory pathways [39,67,68]. Pertaining to HIV, initially, TRIM28 was identified as a restrictor of HIV through its interaction with Integrase, hindering viral integration into the host chromatin [69]. TRIM28 was also shown to inhibit HIV transcription by depleting Tat in the myeloid lineage with the help of CTIP2 [70]. Later, TRIM28 was shown to modulate HIV transcription both positively [67] and negatively [68] by modulating the P-TEFb complex, implicating it in both viral latency and reactivation. Consequently, further investigation is required to delineate the direct or indirect impact of TRIM28 on HIV proviral gene expression. Collectively, these findings [58,60,71] led us to hypothesize that the DNA-PK-mediated phosphorylation of TRIM28 may control a switch between the repression and activation of HIV transcription.

During HIV transcription, the elongation phase of HIV transcription is greatly enhanced by the Tat protein of HIV, as Tat enhances the recruitment of the host cell elongation factor, positive transcriptional elongation factor b (P-TEFb), to the HIV LTR. Subsequently, the CDK9 subunit of P-TEFb catalyzes the hyperphosphorylation of the RNAP II CTD at Ser2 and makes RNAP II processive [49,72]. In addition, CDK9 also catalyzes the phosphorylation of negative factors, namely the DSIF and the NELF, and relieves their negative impact on HIV transcription [73,74]. However, we have shown that CDK9 does not catalyze the phosphorylation of TRIM28 [39]. Our previous studies have shown that the lack of P-TEFb in quiescent primary T cells is responsible for HIV latency, even in the presence of adequate NF-kB activation [75]. The P-TEFb complex consists of other subunits, mainly ELL2, ENL, AFF4, and AF9; together, it is called the super elongation complex (SEC) [76,77,78]. Thus, the HIV Tat protein plays a significant role in augmenting the elongation phase of HIV transcription and generating full-length genomic transcripts of HIV [1,79,80,81]. In the absence of Tat, the elongation or completion of HIV transcripts is inefficient. Once HIV Tat is available, it positively regulates HIV transcription. The process of Tat binding to the trans-activation response (TAR) element, an RNA stem-loop structure of HIV transcript, brings an essential transcriptional component, P-TEFb, thereby enhancing the efficiency of viral transcription by promoting the elongation phase of transcription. HIV transcription auto-accelerates itself by generating more Tat protein [82,83]. Thus, the enhanced rate of HIV transcriptional elongation results in a higher number of complete genomic HIV transcripts and the generation of more viral particles.

In our previous publication, we clarified the important role of the DNA-PK during HIV transcription and documented the continuous presence and gliding of the DNA-PK along with RNAP II across the HIV genome during transcription [37,39]. Additionally, we identified the impact of cocaine use on promoting HIV transcription and replication [16,17,27,28]. Later, we endeavored to define the underlying molecular mechanism through which cocaine augments HIV transcription and found that cocaine promotes HIV transcription by inducing different mechanisms [27,28]. To expand upon this subject, in the present study, we focus on understanding the role of the cocaine-stimulated DNA-PK in relieving RNAP II pausing during HIV transcription by catalyzing TRIM28 phosphorylation selectively at S824 residue. We found that cocaine further enhances the nuclear localization of the DNA-PK, where the DNA-PK facilitates HIV transcription. We noted that cocaine exposure not only augments nuclear localization but also enhances its functional activity by increasing its phosphorylation at a specific residue, Ser2056. Subsequently, we substantiated the finding of increased HIV transcription following cocaine exposure by examining the effect of the cocaine-induced DNA-PK on the phosphorylation of specific sites on the RNAP II CTD, namely Ser2 and Ser5. To further authenticate the precise role of the cocaine-induced DNA-PK in CTD phosphorylation, we investigated the inhibitory potential of clinically evaluated DNA-PK inhibitors in reversing the influence of the DNA-PK. These findings were further validated by conducting DNA-PK knockdown experiments in the presence or absence of cocaine, demonstrating the specific impact of cocaine-induced DNA-PK stimulation. Overall, our data demonstrate the crucial role of cocaine-mediated DNA-PK stimulation in relieving RNAP II pausing by converting TRIM28 from a transcriptional inhibitor to a transcriptional activator (transactivator) protein. These findings were validated across diverse cell types belonging to both lymphoid and myeloid lineages, including microglia, the macrophages that reside in the CNS. This comprehensive study expands our understanding of the complex interplay among cocaine, the DNA-PK, and TRIM28 and their influence on HIV transcription. Consequently, it illuminates potential therapeutic strategies for addressing HIV replication and/or mitigating the toxicities associated with drug abuse. Additionally, given that ART is unable to restrict HIV transcription or latency-reactivation, defining all factors and mechanisms that regulate HIV transcription will help open new avenues for better translational interventions.

## 2. Materials and Methods

### 2.1. Plasmid Construction, Gene Transfer, Transfection, and VSV-G Pseudotyped Virus Generation

A pHR’p-Luc plasmid was constructed by inserting the EcoRI and XhoI fragment of HIV pNL4-3 into a pHR’ plasmid, as detailed previously [84,85]. The procedure for the knockdown of the DNA-PK was also described previously [39]. The short-lived variant of the green fluorescent protein (d2EGFP) was inserted at the nef position using the MluI and XhoI sites. Site-directed mutagenesis was conducted to substitute histidine at position 13 with leucine (H13L) (CAT to TTA), following established procedures [86,87]. Human Embryonic Kidney 293 cells (HEK 293T) were cultured in Dulbecco’s Modified Eagle Medium (DMEM), supplemented with 2.05 mL of glutamine (Hyclone, ThermoScientific, Waltham, MA, USA), 10% fetal bovine serum (Gemini, West Sacramento, CA, USA), and 1 U/mL of penicillin/streptomycin. The cells were seeded, grown to 70% confluency, and rinsed with Opti-MEM I (1×) + GlutaMAX-I Reduced Serum Medium (Gibco, Waltham, MA, USA) before transfection. Transfection was performed by using Lipofectamine 2000 (Invitrogen, Waltham, MA, USA), as per the manufacturer’s instructions. Briefly, 35 µL of Lipofectamine 2000 reagent was mixed with 500 µL of Opti-MEM. Separately, 18 µg of a plasmid DNA mixture (3 µg pCMVΔ8.9.1, 4 µg pMD.G, 3 µg pMDL-g/p-RRE, 1 µg pRSV-Rev, and 7 µg of either pHR’P-Luc or pNL4-3-ΔE-EGFP for generating pNL4-3-ΔE-EGFP and pHR’p-P-Luc pseudotyped viruses, respectively) was prepared [28]. The two solutions were combined and incubated at room temperature (RT) for 30 min to form the lipid–DNA complex, which was then introduced into the cells. Five hours after transfection, the culture medium was replaced with fresh DMEM. The cell supernatant containing the virus was collected at 48 h and 72 h post-transfection.

### 2.2. Generation of Luciferase Cell Line and Latently Infected Jurkat T-Cell Clones

The pHR’p-Luc virus was transduced into the Jurkat cell line via spinoculation in the presence of 8 µg/mL polybrene. Successful infection was subsequently confirmed by a luciferase assay [85]. The isolation of Clone 2D10 cells, characterized by the H13L Tat mutation, was detailed in our previous study [87]. Specifically, Vesicular Stomatitis Virus Protein G (VSV-G)-pseudotyped HIV particles were generated through the triple transfection of 293T cells using Lipofectamine 2000 reagent (Invitrogen, Waltham, MA, USA). Virus titers were determined by infecting 2 × 10^6^ Jurkat T-cells with serial dilutions of the concentrated virus preparation obtained from the harvested medium supernatant. Six hours post-infection, the cells were rinsed with phosphate-buffered saline (PBS), and the RPMI 1640 medium was replenished. The expression of d2EGFP was assessed by fluorescently activated cell sorting analysis (FACS Calibur, NJ, USA) 72 h post-infection, and d2EGFP expression was subsequently analyzed every week until the cells were fully shut down without detectable d2EGFP expression before reactivation experiments.

### 2.3. Cell Culture and Cell Experiments

Microglial, THP-1, MT-4, peripheral blood mononuclear cells (PBMCs), Jurkat, and derivatives of Jurkat cells (Clone 2D10 and Jurkat-pHR’P-Luc) were cultured in either DMEM or RPMI 1640 medium. The culture medium was supplemented with 10% fetal bovine serum (FBS), penicillin (100 IU/mL), streptomycin (100 IU/mL), and 25 mM of HEPES. The cells were maintained at 37 °C in a 5% CO_2_ environment. Fresh medium was replenished every 2–3 days, and cell density was kept at 2 × 10^6^ cells/mL.

### 2.4. HIV Replication-Competent Virus

The Human Immunodeficiency Virus Type 1 (strain 93/TH/051) was obtained from the National Institute of Health AIDS reagent program. Primary HIV isolates were cultured following the instructions provided in the datasheet obtained through the UNAIDS Network for HIV Isolation and Characterization. Briefly, 4 × 10^6^ stimulated Jurkat cells (cells previously stimulated with PHA for 4 days and treated with polybrene) were collected and exposed to HIV (strain 93/TH/051) for 30 min at 37 °C. Following this, fresh media was added, and the cells were incubated for 5 days. The cell-free virus was recovered, aliquoted in multiple stocks, and stored at −80 °C until use.

### 2.5. Cocaine Treatment and Inhibitor Treatment

Cocaine hydrochloride was obtained from the National Institute on Drug Abuse (NIDA) Drug Supply Program. In this study, various cocaine concentrations were employed. Nonetheless, the maximum concentration utilized was 30 µM cocaine, which falls below the levels typically observed in the plasma of human drug users. All cocaine treatments were conducted at a concentration of 10 μM unless otherwise specified. Acute treatment involved exposing the cells to cocaine for 3 h, whereas chronic treatment entailed exposing the cells to cocaine twice daily for two consecutive days, with an additional 3 h exposure prior to cell harvesting. The inhibitors (M3814 and NU7441) were treated overnight (24 h) prior to exposure to cocaine.

### 2.6. Infection of Cells with Replication-Competent Virus

The cells (5 × 10^6^ cells) were either untreated or exposed to cocaine for 3 h in the presence or absence of M3814 and, subsequently, were either uninfected or infected with the replication-competent virus (1 mL) for 24 h and 48 h to assess HIV gene expression. The inhibitors were administered 24 h before HIV infection, with the specific doses mentioned in the figure legends.

### 2.7. Western Blot Analysis of Total Cell Lysate

The cells (1 × 10^6^ or 5 × 10^6^ cells approx.) were treated with cocaine in the presence or absence of M3814 (the DNA-PK inhibitor) and/or infected with the replication-competent virus for 24 h and 48 h. Subsequently, the samples were collected and washed with 1 mL of ice-cold PBS, and 100 µL of 1X passive lysis buffer (Promega, Madison, WI, USA) was added to the cells. The cell lysate with the lysis buffer was then incubated on ice for 30 min. During the incubation, cells were vortexed for 30 s for complete lysis after every 10 min. Following incubation, the cell lysate was centrifuged at the highest speed for 30 min, and the supernatant was analyzed for protein concentration using a Pierce™ BCA Protein Assay Kit. The protein concentration was normalized, and an equal amount of protein was mixed with 5× Laemmle Sample buffer, heated to 95 °C for 10 min, and then resolved by SDS-PAGE on a 6% or 10% or 12% gel at 120 volts until the dye reached the bottom. The resolved proteins were then transferred to a nitrocellulose membrane. The membranes were blocked with 3% bovine serum albumin (BSA) for 1 h and incubated with primary antibodies at 4 °C overnight and then with secondary antibodies (1:15,000 dilution) for 1 h at room temperature. After three washes with 1× TBST, the blot was detected using the Odyssey infrared imaging system application software version 3.0 (LI-COR Biosciences, Lincoln, NE, USA).

### 2.8. Western Blot Analysis of Cytoplasmic and Nuclear Extracts

The cells (5 × 10^6^ or 1 × 10^7^ cells approx.) were exposed to cocaine at various doses and time points, with or without the inhibitor. Subsequently, the cells were collected and washed with 1 mL of ice-cold PBS. Following our established protocol, we fractionated cytosolic and nuclear proteins. Initially, the cells were allowed to swell in 200 µL–500 µL of cytoplasmic extract (CE) buffer (1 mM Hepes KOH pH 7.9, 60 mM KCl, 1 mM EDTA, 0.5% NP-40, 1 mM DTT, and 1 mM PMSF) for 10 min on ice, during which cells were vortexed for lysis. The nuclei were then pelleted at 4000 r.p.m for 10 min. The cytoplasmic lysates were transferred to new Eppendorf tubes for analysis of cytoplasmic proteins. The nuclei were washed twice with 1 mL of CE buffer, pelleted at high-speed centrifugation for 2 min, and subsequently resuspended in 80 µL of nuclear extract (NE) buffer (250 mM Tris pH 7.8, 60 mM HCl, 1 mM EDTA, 1 mM DTT, and 1 mM PMSF). The nuclei were lysed through 8 freeze-thaw cycles in liquid nitrogen. The nuclear lysate was cleared through centrifugation at high speed for 1 min, and the supernatant was transferred into a new microfuge tube. The total nuclear protein concentration in the samples was normalized using a standard BCA assay. An equal amount of the total nuclear samples was loaded and resolved by 6%, or 10%, or 12% SDS-PAGE gel for electrophoresis. The proteins on the gels were transferred onto nitrocellulose membranes; blocked with 3% BSA for an hour; incubated with primary antibodies overnight and with secondary antibodies for an hour; and finally detected using the Odyssey infrared imaging system application software version 3.0 (Li-cor Bioscience).

The actin was selected as the loading control to ensure equal protein loading across all the wells. Actin’s presence in equal amounts among samples confirms uniform loading, irrespective of the sample’s cytoplasmic or nuclear nature. However, to confirm the presence of an exclusive nuclear protein fraction, we used HDAC1, a protein that exclusively resides in the nucleus.

### 2.9. Chromatin Immunoprecipitation (ChIP) Assay

The ChIP assays were performed using our well-established protocol [75,85]. Briefly, 1 × 10^8^ freshly infected cells with replication-incompetent HIV (pHR’P-Luc) were exposed to cocaine. Later, the cells were treated with 0.5% formaldehyde for 10 min with rotation at room temperature, facilitating the crosslinking of proteins to DNA. Glycine was added to stop the crosslinking process. The cells were harvested, washed twice with ice-cold PBS, and allowed to swell for 10 min in 5 mL CE buffer. The nuclei were pelleted after centrifugation at 4000 rpm for 10 min and resuspended in 1 mL of SDS Lysis buffer (50 mM Tris-HCl, 1% SDS, 10 mM EDTA, 1 mM PMSF, 1 µg/mL aprotinin, and 1 µg/mL pepstatin A). The genomic DNA was fragmented to lengths less than 800 bp by sonication (Misonix 3000, NY, USA) under the following conditions: output 2.5 for 20 s, repeated eight times. For each sample, 200 µL of the sonicated samples were mixed with 800 µL of ChIP dilution buffer (0.01% SDS, 1.1% Triton X-100, 1.2 mM EDTA, 16.7 mM Tris-HCl pH 8.1, and 167 mM NaCl). The samples were incubated with specific antibodies including DNA-PKcs, RNAP II, CDK7, CDK9, pTRIM28 (S824), and H3K27me3 at +4 °C overnight. Protein A/G Sepharose was pre-saturated with salmon sperm DNA and 1% BSA, and 100 µL of 25% Protein A-Sepharose was utilized in DNA-protein immunoprecipitation. Following 3 h of incubation, antibody–DNA–protein complexes were washed with 1 mL of each washing buffer. The first wash occurred with a low salt immune complex wash buffer (0.1% SDS, 1% Triton X-100, 2 mM EDTA, 20 mM Tris-HCl pH 8.1, and 150 mM NaCl), followed by a high salt immune complex wash buffer (0.1% SDS, 1% Triton X-100, 2 mM EDTA, 20 mM Tris-HCl pH 8.1, and 500 mM NaCl). The complexes underwent further washing with lithium chloride buffer (0.25 M LiCl, 1% NP-40, 1% sodium deoxycholate, 1 mM EDTA, and 10 mM Tris HCl pH 8.0) and were washed twice with TE buffer (10 mM Tris-HCl pH 8.0, 1 mM EDTA pH 8.0). Protein DNA complexes were eluted from protein A/G Sepharose twice using 250 µL of freshly prepared elution buffer (1% SDS and 0.1 mM NaHCO_3_). Twenty microliters of 5 M NaCl were added to the total eluate, and the protein–DNA complexes were reverse-cross-linked at 65 °C overnight. Ten microliters of 0.5 M EDTA, 10 µL of 2 M Tris-HCl pH 6.5, and 2 µL of 10 ng/mL proteinase-K were added, and samples were incubated at 50 °C for 2 h followed by phenol extraction and ethanol precipitation. The precipitated DNA samples were dissolved in 100 µL of TE buffer, and 2 µL of the sample was utilized in real-time PCR using an SYBR green PCR master mix (Thermo Scientific), following the method described previously by Kim et al. [51]. The without-antibody control values were subtracted from each sample value to eliminate the non-specific background signal. The primer sets utilized in real-time PCR amplification are listed in Supplementary Table S1.

### 2.10. RNA Extraction and Real-Time Quantitative PCR (qPCR)

The total RNAs were extracted from 5 × 10^5^ cultured cells using an RNA isolation kit (Qiagen, Hilden, Germany) according to the manufacturer’s instructions. The isolated RNAs were meticulously assessed for their integrity, purity, and yield. Subsequently, using the isolated RNAs as the template, the first-strand complementary DNA (cDNA) was synthesized utilizing M-MLV Reverse Transcriptase (Thermo Scientific, Waltham, MA, USA). In brief, approximately 3 µg of the extracted RNAs were reverse transcribed in a total volume of 20 µL with 350 µM dNTP, 50 µM of oligo (dT), 5× M-MuLV buffer, 200 U RNase inhibitors, and 200 U M-MuLV reverse transcriptase. The RNA, oligo (dT), and dNTPs were mixed and incubated at 65 °C for 5 min, followed by 37 °C for 50 min and 70 °C for 10 min. The cDNA was subsequently diluted and subjected to real-time PCR using the Real-Time PCR system 7500TH (Life Technologies, Carlsbad, CA, USA). For all samples, actin/GAPDH was measured as the internal control and utilized for data normalization. The primer sets utilized for the amplification are listed in Supplementary Table S1.

### 2.11. Luciferase Assay

The 1 × 10^4^ or 5 × 10^5^ cells harboring pHR’P-Luc were plated in 12-well plates with complete RPMI media (supplemented with 10% FBS, penicillin, and streptavidin). The cells were incubated with cocaine (chronically, treated twice per day with cocaine) for 48 h in the presence and absence of M3814. Luciferase levels in the cells were assessed using a Luciferase Assay System kit (Promega, Madison, WI, USA). Briefly, the cells were harvested, washed, and lysed with 1× passive lysis buffer. After incubating for 30 min at RT, cells were centrifuged at high speed for 2 min, and the supernatant was transferred to a new Eppendorf tube. The total protein concentration of each sample was determined using standard BCA assays. The equal amounts of protein among samples were used to perform luciferase assays. A total of 10 µL of each sample lysate was added followed by 50 µL of luciferase substrate/assay buffer to individual wells of white plates to reflect light and maximize the light output signal. Each sample was tested in triplicate. Luminescence was read in a Veritas Microplate Luminometer (Turner Biosystems, Sunnyvale, CA, USA).

### 2.12. Flow Cytometry (FACS) Analysis

FACS analyses were performed on 2D10 cells (Jurkat cells infected with the VSV-G pseudotyped HIV virus carrying the GFP gene under the control of the HIV LTR promoter). Briefly, 2D10 cells were treated with inhibitor M3814 for 24 h. The next day, cells were activated/stimulated with 10 ng/mL of Tumor Necrosis Factor alpha (TNF-α) for another 48 h. Cells were then harvested, washed, re-suspended with PBS, and analyzed with a FACS Calibur (BD Biosciences, Franklin Lakes, NJ, USA) using FlowJo software (Treestar Inc., Ashland, OR, USA, version 10.10).

### 2.13. Immunofluorescence Assay (IFA)

After cocaine treatment, cells were fixed with 4% paraformaldehyde (PFA) for 15 min at RT. Subsequently, the cells were permeabilized with 0.25% Triton X-100 for 10 min at RT and blocked using a blocking solution containing 10% horse serum and 2% bovine serum albumin (BSA) for 1 h at RT. The cells were then incubated with antibodies against the pDNA-PKcs (Ser2056) and the DNA-PKcs (E-6) overnight at 4 °C. The following day, unbound antibodies were removed through washing, and the cells were incubated with fluorescently conjugated secondary antibodies for 45 min. Cells were stained with DAPI at room temperature for 15 min, washed three times, and then mounted on a slide using the Aqua Mount mounting medium (Epredia 13800, Dearborn, MI, USA). Imaging was performed using a confocal microscope (Nikon A1R, Tokyo, Japan) with either a 20× or 60× objective and Z-stack images were acquired to create composite 3D images.

### 2.14. Quantification and Statistical Analysis

Data are expressed as the mean standard deviation (mean ± SD). Comparisons between the two groups were performed using Student’s *t*-test. Comparisons between more than two groups were carried out by one-way or two-way analysis of variance (ANOVA). If the *p*-value obtained from ANOVA was under 0.05 (*p* < 0.05), it was considered statistically significant. All statistical calculations were carried out using a GraphPad prism (Ver 9.4.0). All the statistical details of experiments can be found in the figure legends.

## 3. Results

### 3.1. Cocaine Enhances Both the Catalytic Activity and the Nuclear Localization of the DNA-PK

**Figure 1:**
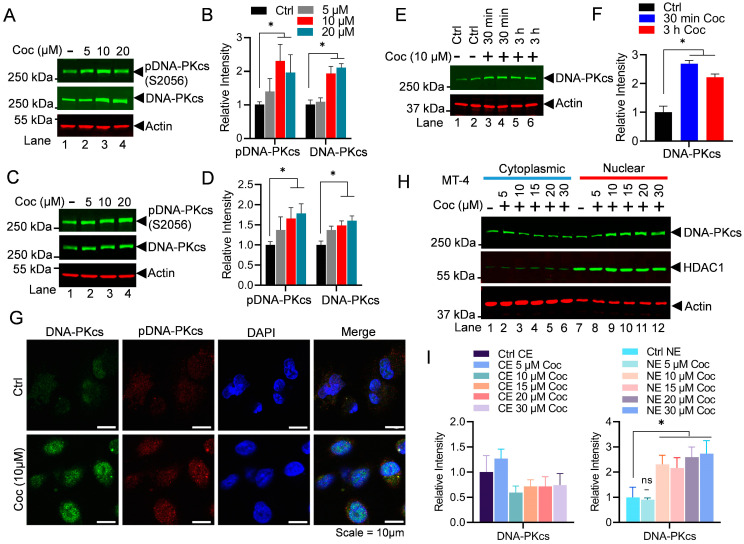
Cocaine enhances both the catalytic activity and nuclear level of the DNA-PK. MT-4 (**A**) and microglial cells (**C**) were treated with different concentrations of cocaine (Coc: 5, 10, and 20 μM) for 3 h (Lanes 2 to 4). Jurkat-pHR’-P’-Luc cells were treated with 10 µM cocaine (Coc) in replicates for 30 min and 3 h (Lanes 3 to 6) (**E**). Cells were harvested, and nuclear lysates were analyzed by immunoblotting using specific antibodies, pDNA-PKcs (S2056) and the DNA-PKcs, as indicated. Actin, a constitutively expressed protein, was used as a loading control. Densitometric analysis of protein bands (normalized to actin) confirmed the significant upregulation of the total DNA-PKcs and its phosphorylated form, pDNA-PKcs S2056 (pDNA-PKcs), following cocaine treatment (**B**,**D**,**F**). Representative immunofluorescence images (**G**), show control (Ctrl) and cocaine-treated (Coc) cells probed with anti-DNA-PKcs (Green) or anti-pDNA-PKcs (2056) (Red) antibodies which are co-labeled with DAPI (blue) for nuclear visualization. Images were captured at 60× magnification; the scale bar represents 10 µM (**G**). In panel (**H**), the MT-4 cells were treated with increasing concentrations of cocaine for 3 h. Cells were harvested and lysed, and both cellular and nuclear lysates were analyzed by immunoblotting with antibodies against DNA-PKcs, HDAC1, and actin (**H**). Densitometric analysis of the cellular and nuclear DNA-PKcs upon cocaine treatment (**I**). Immunoblots shown are representative of at least three independent experiments. The results are expressed as the mean ± SD and were analyzed by one- or two-way ANOVA, followed by Tukey’s multiple comparison test. Asterisks over the bars indicate significant differences: * *p* < 0.05 for the comparisons of cocaine-treated cells vs. untreated cells (Ctrl).

The crucial role of the DNA-PK during DNA double-strand break repair is well established [43,44,45]. However, for the first time, we documented the vital role of the DNA-PK in supporting gene transcription [37]. To define the underlying molecular mechanism through which the DNA-PK augments HIV transcription, we confirmed that the DNA-PK augments HIV transcription by supporting both the initiation and elongation phases of transcription [46]. Later, the crucial role of the DNA-PK in supporting other cellular genes by enhancing RNAP II CTD phosphorylation was confirmed by us and others [39,58,59,60]. Previously, we identified the significant impact of cocaine on enhancing HIV transcription and replication [27,28]. However, to develop therapeutic strategies aimed at mitigating the toxic effects resulting from HIV replication and cocaine exposure, it is imperative to elucidate all the factors and mechanisms through which HIV and cocaine collaborate to induce cell toxicity via heightened HIV transcription. To investigate the role of cocaine in enhancing HIV transcription, we assessed the expression and nuclear level of the DNA-PKcs, the catalytic subunit of the DNA-PK. The impact of cocaine on the functional/catalytic activity of the DNA-PK was evaluated by examining the phosphorylation of the pDNA-PKcs at serine 2056 (p-DNA-PK S2056), a post-translational modification that marks the functionally active form of the DNA-PK. We treated MT-4 cells, a T cell line, with increasing doses of cocaine for a duration of 3 h. Later, cells were harvested, and nuclear lysates were subjected to immunoblotting using antibodies specific for either the total DNA-PKcs or the phosphorylated form of DNA-PKcs (pDNA-PKcs S2056) to evaluate cocaine impact. We found higher levels of both the DNA-PKcs and pDNA-PKcs S2056 in the nucleus upon cocaine exposure compared to the untreated cell control (Ctrl) (Figure 1A,B). The densitometric analyses of the protein bands validated a significant increase in the expression and nuclear level of both the DNA-PKcs and pDNA-PKcs S2056 following cocaine-mediated cell stimulation. We further confirmed the effect of cocaine in upregulating and activating the DNA-PK in a dose-dependent manner using other varying cell types belonging to different lineages, namely, microglial cells, a primary immune cell found in CNS (Figure 1C,D). These findings confirmed the significant upregulation of DNA-PK expression and functional activation of the DNA-PK (pDNA-PKcs S2056) by cocaine and in a cell lineage-independent manner.

Subsequently, we examined the impact of cocaine on DNA-PK levels and activation in a time-dependent manner (Figure 1E,F) by treating the Jurkat cells infected with pHR’P-Luc with a fixed dose of cocaine (10 μM) for 30 min and 3 h, with untreated cells as a control (Figure 1E,F). Upon analyzing the nuclear extract, we found the upregulation of nuclear DNA-PK levels within 30 min, which remained higher even after 3 h.

Further, we performed an immunofluorescence assay to visualize the nuclear level of the DNA-PK. We treated the microglial cells with cocaine for 3 h, then fixed them with 4% paraformaldehyde (PFA). After fixing, cells were permeabilized and incubated with primary antibodies, pDNA-PKcs (S2056), and the DNA-PKcs (total) followed by corresponding fluorescent secondary antibodies, and were finally visualized under a confocal microscope. Corroborating to the above immunoblotting results (Figure 1A–F), our immunofluorescence data reveal the significant upregulation of both nuclear DNA-PKcs and pDNA-PKcs (S2056) levels following cocaine treatment (Figure 1G and Supplementary Figure S1). Notably, the increased nuclear localization of the DNA-PK has been previously observed in response to cellular stimulation [38], further supporting our findings.

Furthermore, to validate the findings, MT-4 cells were treated with increasing doses of cocaine for 3 h, and the protein levels of the DNA-PKcs in the cytoplasm and the nucleus were evaluated by immunoblotting. We quantified and loaded equal amounts of both cytosolic and nuclear protein on the SDS-PAGE gel. In the same gel, both cytoplasmic and nuclear protein fractions were analyzed. As a control, we evaluated HDAC-1 levels, a protein that predominantly exists in the nucleus, and only a small portion was present in the cell cytoplasm. Accordingly, we found the abundant presence of HDAC-1 in the nuclear extract of the cell, validating the purity of our nuclear fraction and our assay conditions. As a loading control, we examined the presence of the Beta-actin protein, which is constitutively expressed in the cell and can be detected in both cytoplasmic and nuclear fractions. Interestingly, we noted significantly enhanced nuclear levels of the DNA-PKcs following cocaine treatments (Figure 1H,I). These results demonstrate that cocaine enhances DNA-PK function by both upregulating its expression and promoting its nuclear localization, while also increasing its catalytic activity through specific phosphorylation at S2056. The elevated nuclear levels of the DNA-PK following cocaine exposure strongly suggest its involvement in DNA-related processes, including transcription. Altogether, these findings confirm that cocaine intake promotes the activation of the DNA-PK by augmenting both its nuclear localization and functional activity.

### 3.2. Cocaine-Induced HIV Transcription Augments Overall HIV Replication

**Figure 2:**
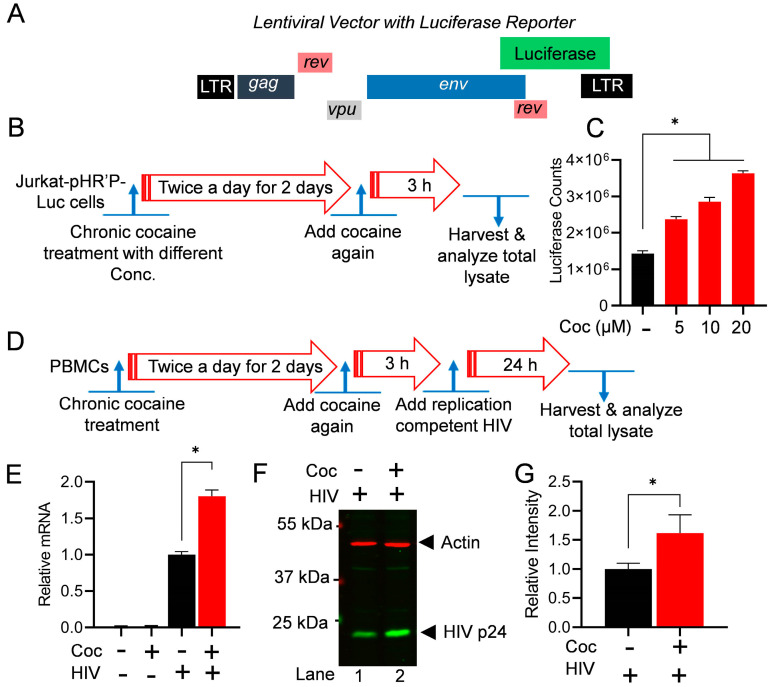
Cocaine-induced HIV transcription augments overall HIV replication. The structure of the lentiviral vector (pHR’-PNL-Luc) carrying the reporter luciferase gene under the HIV LTR promoter (**A**). A schematic representation of the cocaine (Coc) treatment scheme to perform a luciferase reporter assay (**B**). Jurkat-pHR’-P-Luc cells were chronically treated with 5 µM–20 µM of cocaine. The cells were lysed, the protein was quantified, and an equal amount of protein was used in each sample to perform luciferase assays (**C**). A schematic depiction of the cocaine treatment and subsequent infection of PBMCs with replication-competent HIV (**D**). HIV transcripts were quantified by real-time PCR using primer sets that amplify the Envelope (Env) region of the HIV genome (**E**). The level of Gag/p24 protein was analyzed by immunoblotting with specific antibodies against HIV p24 (**F**). Actin, a constitutively expressed protein, was used as a loading control in the same blot. Densitometric analysis of protein bands (normalized to actin) confirmed a significant increase in p24 levels compared to untreated cells (Ctrl) (**G**). Immunoblots are representative of at least three independent experiments. The black bar in the graphical representation represents the control, while the red bar indicates the presence of the cocaine. The results are expressed as the mean ± SD, analyzed by one-way ANOVA followed by Tukey’s multiple comparison test (**C**,**E**) or unpaired *t*-test (**G**). Asterisks over the bars indicate significant differences: * *p* < 0.05 for the comparison of cocaine-treated cells vs. untreated control cells.

To evaluate the impact of cocaine on HIV transcription and subsequently on HIV gene expression, we freshly infected Jurkat cells with non-replicating attenuated HIV, pHR’P-Luc, to generate the Jurkat-pHR’P-Luc cell line [85]. The pHR’P-Luc is an HIV-based lentivirus that expresses the luciferase reporter gene under the control of the HIV LTR promoter (Figure 2A). Therefore, the expression of luciferase indicates ongoing HIV transcription and gene expression. Figure 2B,D depict the schematic overview of the cell treatment procedures. As anticipated from our previous studies [27,28], a significant increase in luciferase counts was observed in a dose-dependent manner, validating the cocaine-mediated upregulation of HIV gene expression (Figure 2C). To further confirm the impact of cocaine-mediated cell stimulation on HIV gene expression and replication, PBMCs were chronically treated with cocaine prior to being infected with a replication-competent dual tropic HIV Type 1 strain 93/TH/051 for a period of 24 h. The HIV transcripts were quantified via real-time qPCR using primer sets that amplify the Envelope (Env) region of the HIV genome. A significant upregulation of HIV gene expression was confirmed in the presence of cocaine (Figure 2E). Next, the HIV protein expression was evaluated via immunoblotting using antibodies against Gag subunits (p24) of HIV by comparing the cell lysates of cocaine-treated or untreated HIV-infected cells (Figure 2F,G). The upregulation of p24 confirms enhanced HIV gene expression and replication in the presence of cocaine. Together, these results suggest that cocaine-induced signaling pathways promote the activation of both the cell status and transcription machinery, including DNA-PK stimulation (p-DNA-PK S2056) (Figure 1), resulting in enhanced HIV transcription and consequently higher HIV replication.

### 3.3. Partial DNA-PK Inhibition Is Sufficient to Restrict HIV Transcription, Replication, and Latency Reactivation

We have shown that the DNA-PK plays an important role in HIV transcription [37,39]. To extend further on these findings and establish the translational potential of DNA-PK inhibition in restricting HIV transcription and replication, we evaluated the role of a clinically evaluated DNA-PK inhibitor (DNA-PKi), M3814. Interestingly, in a recent clinical study, DNA-PK inhibitors, including M3814 at dosages from 110 µM to 320 µM, were found to be safe and highly effective as potential anti-cancer drugs [88,89,90,91,92,93,94,95,96,97,98], validating the safety of these agents for human use [90]. Notably, we found that partial DNA-PK inhibition by only 20 µM (less than 1/5) is sufficient to restrict HIV transcription, replication, and latency reactivation without any cell toxicity.

**Figure 3:**
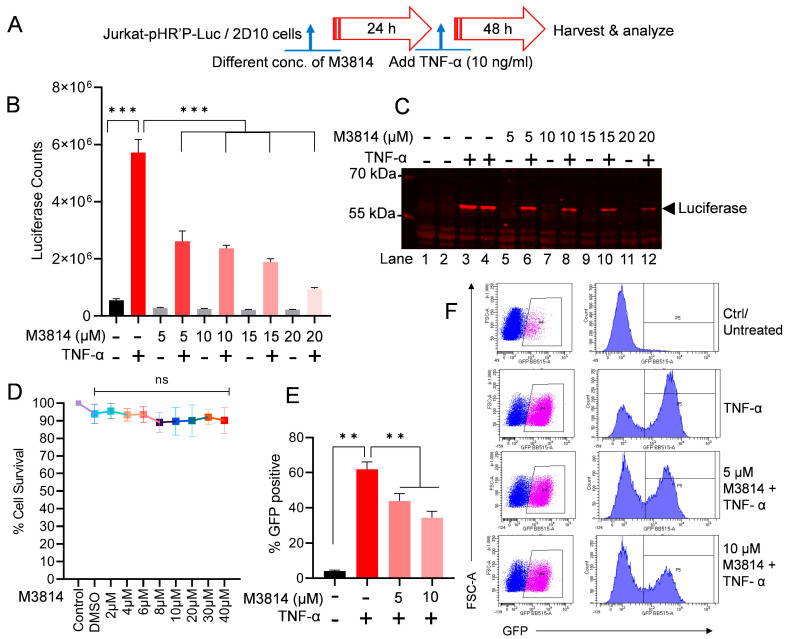
Partial DNA-PK inhibition severely impairs HIV transcription and latency reactivation. A schematic representation of the protocol for the luciferase reporter assay detailing treatment with the DNA-PK inhibitor (DNA-PKi), M3814, and TNF-α (**A**). Jurkat-pHR’-P-Luc cells were treated with 5, 10, 15, and 20 µM of M3814 for 24 h, followed by activation with TNF-α (10 ng/mL) for another 48 h. Cells were lysed, the cell extract was quantified, and an equal amount of protein per sample was used to perform luciferase assays (**B**). The equal amounts of the same lysates were analyzed by immunoblotting using specific antibodies against the luciferase protein (sc-74548) (**C**). Jurkat-pHR’-P-Luc cells were cultured with different concentrations (2 μM to 40 μM) of M3814 for 48–72 h, and cell cytotoxicity was determined via MTS-PMS cell proliferation assay (Promega, Madison, WI, USA) (**D**). Latently infected 2D10 cells, which express a short-lived green fluorescent protein (d2EGFP) under the control of the HIV LTR promoter, were treated with 5 µM or 10 µM of M3814 for 24 h. Later, cells were stimulated with TNF-α for an additional 48 h. GFP expression was then assessed using flow cytometry (**E**,**F**). Immunoblots shown are representative of at least three independent experiments. In the graph, the black bar represents control, the gray bar denotes the absence of TNF-α, the red bar shows the presence of TNF-α alone, and fading red colors indicate dose-dependent inhibition of HIV by the inhibitor in presence of TNF-α. The results are expressed as the mean ± SD and analyzed by one- or two-way ANOVA followed by Tukey’s multiple comparison test. “ns” indicates not significant. Asterisks over the bars indicate significant differences: ** *p* < 0.01 and *** *p* < 0.001 for the comparison of either inactive vs. activated cells (TNF-α) or activated cells (TNF-α) vs. activated cells (TNF-α) treated with the DNA-PKi, M3814.

We assessed the effect of M3814 on HIV transcription and latency reactivation. The infected Jurkat cells that harbor latent HIV provirus (pHR’P-Luc) in their genome, which expresses the luciferase reporter gene under the control of the HIV LTR promoter were used (Figure 2A). These cells, Jurkat-pHR’P-Luc, were incubated overnight (24 h) with increasing concentrations (5 µM, 10 µM, 15 µM, and 20 µM) of M3814. The next day, the cells were stimulated with 10 ng/mL Tumor Necrosis Factor alpha (TNF-α) for another 48 h (Figure 3A). A strong M3814-mediated dose-dependent inhibition of HIV transcription was observed, indicated by highly reduced luciferase counts, marking restricted HIV gene expression when the DNA-PK was selectively inhibited using a highly specific and clinically evaluated DNA-PKi (Figure 3B). As controls, cells were either treated with TNF-α alone (positive control) or left untreated (negative control). The inverse correlation between luciferase counts and M3814 concentration confirms the direct role of the DNA-PK in supporting HIV transcription and latency reactivation (Figure 3B). These findings were further validated by examining the presence of the luciferase protein in the cell extracts by performing immunoblotting using an antibody specific to the luciferase protein (luciferase antibody: sc-74548) (Figure 3C). The strong dose-dependent inhibition of luciferase by M3814 established the vital role of the DNA-PK during HIV transcription. Overall, these findings demonstrate the pivotal role of the DNA-PK in supporting HIV transcription and latency reactivation. Moreover, the data obtained confirm our previous findings where we used another highly specific clinically tested DNA-PKi (Nu7441) [39].

To exclude the possibility that the reduced luciferase activity upon M3814 treatment was due to cell loss, we performed a cell viability assay. The Jurkat-pHR’P-Luc cells were cultured with different concentrations (2 μM–40 μM) for M3814 for 48–72 h, and cell cytotoxicity was determined by an MTS-PMS cell proliferation assay (Promega, Madison, WI, USA). We did not observe any significant cell cytotoxicity, even at 40 μM of M3814 treatment (Figure 3D).

The impact of M3814 in restricting the reactivation of latent HIV was further confirmed using another latently infected cell line, 2D10 cells. The 2D10-cell line is a latently infected Jurkat T-cell line, which harbors a latent HIV provirus in their genome that expresses a reporter short-lived green fluorescent protein (d2EGFP) from the HIV LTR promoter [85,87]. Thus, GFP expression marks ongoing HIV gene expression. The 2D10 cells were treated for 24 h with different doses of M3814. The next day, cells were activated with 10 ng/mL of TNF-α for another 48 h. Later, we quantified GFP expression through flow cytometric analysis. TNF-α, which we used as a positive control, was able to stimulate latent HIV in more than 70% of cells compared to the control (unstimulated cells), marked by GFP expression in most (70%) cells. As anticipated, we observed a clear dose-dependent inhibition of HIV proviral reactivation upon DNA-PK inhibition, indicated by the reduced GFP expression in cells treated with the M3814 in a dose-dependent manner compared to the positive control (TNF-α treated) (Figure 3E,F). Overall, these data suggested that the DNA-PK-mediated stimulation of HIV transcription is required for the reactivation of latent HIV provirus.

**Figure 4:**
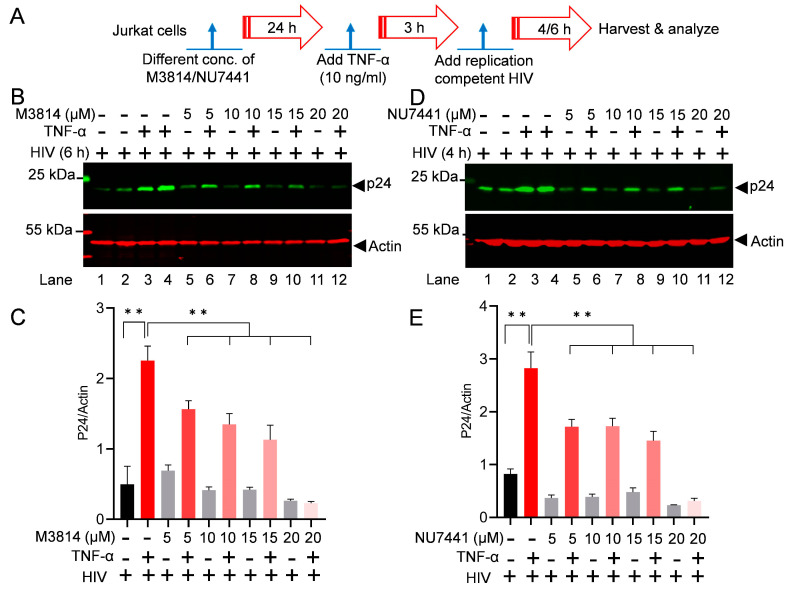
Partial DNA-PK inhibition restricts HIV replication. A schematic representation of the treatment schedule of cells with DNA-PK inhibitors (M3814, NU7441), and/or TNF-α in the presence of HIV infection (**A**). Jurkat cells were treated overnight with either different concentrations of M3814 (5, 10, 15, and 20 μM) (**B**) or NU7441 (5, 10, 15, and 20 µM) (**D**) (Lanes 5–12). The next day, cells were activated with 10 ng/mL TNF-α for 3 h (Lanes 3, 4, 6, 8, 10, and 12). Subsequently, cells were infected with replication-competent dual-tropic HIV (Type 1 strain 93/TH/051) (Lanes 1–12). Cell lysates were prepared 4 h (NU7441) or 6 h (M3814) post-infection (hpi). The cell lysates were quantified, and equal amounts were analyzed per sample via immunoblotting using specific antibodies against HIV gag/p24 protein, as indicated (**B**,**D**). Immunoreactive proteins were detected using appropriately labeled secondary antibodies with Licor. Actin was used as a loading control. Densitometric analysis of protein bands relative to actin (**C**,**E**). Immunoblots are representative of at least three independent experiments. In the graph, the black bar represents control, the gray bar denotes the absence of TNF-α, the red bar shows the presence of TNF-α alone, and fading red colors indicate dose-dependent inhibition of HIV by the inhibitor in presence of TNF-α. The results are expressed as the mean ± SD and analyzed by one-way ANOVA followed by Tukey’s multiple comparison test. Asterisks over the bars indicate significant differences: ** *p* < 0.01 for the comparison of inactive vs. activated cells (TNF-α) and activated cells (TNF-α) vs. activated cells (TNF-α) treated with DNA-PK inhibitors, NU7441 or M3814.

To assess the impact of different highly specific and clinically evaluated DNA-PK inhibitors on HIV replication, Jurkat cells were treated with increasing doses of different DNA-PK inhibitors, M3814 and NU7441, for 24 h. The next day, cells were activated with 10 ng/mL of TNF-α for 3 h. Later, cells were infected with a replication-competent dual tropic HIV (Type 1 strain 93/TH/051). The cell lysates were prepared either 4 h post-infection (4 hpi) or 6 h post-infection (6 hpi), as shown in the figure (Figure 4A). The lysates were analyzed by immunoblotting with the HIV p24 antibody. The results show a clear inhibition of HIV protein expression (HIV p24) with increasing doses of DNA-PK inhibitors M3814 (Figure 4B,C) and NU7441 (Figure 4D,E). The stronger suppression of HIV replication was noted with the increasing doses of DNA-PK inhibitors, indicating target-specific inhibition and confirming the vital role of DNA-PK-induced HIV transcription in supporting overall HIV replication. Additionally, the data confirm that in the presence of the DNA-PKi, TNF-α-mediated strong cell stimulation and NF-kB activation is ineffective in inducing HIV transcription, which suggests that not only cocaine but also TNF-α/NF-kB-mediated HIV transcription requires a functional DNA-PK.

### 3.4. DNA-PK Inhibition Strongly Suppresses Cocaine-Induced HIV Transcription and Replication in Different Cell Types, Including Primary Cells

**Figure 5:**
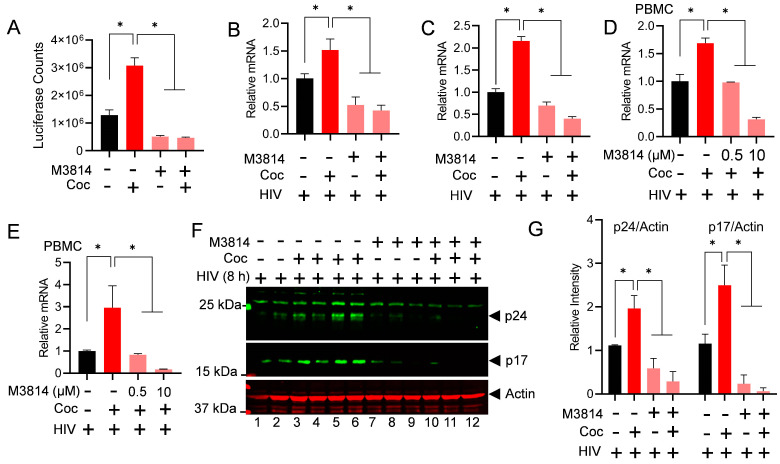
Cocaine-mediated DNA-PK activation promotes HIV transcription and replication in both cell lines and primary cells. Jurkat-pHR’-P-Luc cells were treated with 10 µM of M3814 for 24 h. The next day, cells were treated with cocaine twice daily for 48 h and again for 3 h before harvesting. Cells were lysed, quantified protein amount, and the level of reporter protein expression was determined using a luciferase reporter assay (**A**). Jurkat cells (**B**,**C**) and PBMCs (**D**,**E**) were treated with 10 µM of M3814 for 24 h, then treated with cocaine for 3 h, and subsequently infected with replication-competent HIV for another 3 to 6 h. HIV transcripts were quantified by real-time PCR using primer sets that amplify the Nuc-2 (**B**,**D**) and Env (**C**,**E**) regions of the HIV genome. In panel (**F**), Jurkat cells were treated with 10 µM of M3814 for 24 h (Lanes 7 to 12), then treated with cocaine for 3 h (Lanes 3–6 and 10–12), and infected with replication-competent HIV across all lanes (Lanes 1–12) for another 5 h. The levels of HIV p24 and p17 proteins were analyzed via immunoblotting using antibodies against those proteins (**F**). Actin, a constitutively expressed protein, was used as a loading control. Densitometric analysis of protein bands (normalized to actin) was performed (**G**). Immunoblots are representative of at least three independent experiments. In the graph, the black bar represents the control, the red bar indicates the presence of cocaine, and the fading red colors show the effect of the inhibitor, with or without cocaine. The results are expressed as the mean ± SD and analyzed by two-way ANOVA followed by Tukey’s multiple comparisons test. Asterisks over the bars indicate significant differences: * *p* < 0.05 for the comparison of cocaine-treated samples vs. untreated (Ctrl) and the comparison of cocaine plus inhibitor-treated samples vs. cocaine alone-treated samples.

The above data and our previous publication suggest that cocaine plays a significant role in enhancing HIV transcription and replication [27,28]. In order to understand the molecular mechanisms by which cocaine controls HIV transcription and gene regulation, we investigated whether cocaine promotes HIV transcription and replication by enhancing both the catalytic activity and nuclear level of the DNA-PK. To test this hypothesis, we treated the cells infected with pHR’P-Luc, which carries proviral HIV that expresses the luciferase reporter under the HIV LTR promoter, with 10 µM M3814 for 24 h. The next day, cells were treated with cocaine chronically for two days (10 µM of cocaine twice a day for 3 days). Later, the cell extracts were prepared and quantified, and the level of luciferase reporter protein expression was determined via luciferase assays. As anticipated from the above analysis (Figure 2B), we noticed a significant upregulation of luciferase counts, indicating enhanced HIV transcription in cocaine-treated samples. However, in the presence of M3814, HIV transcription was strongly restricted both in the presence and absence of cocaine (Figure 5A). These results confirmed the specific role of the cocaine-stimulated DNA-PK in promoting HIV transcription and gene expression. Subsequently, to assess the effect of cocaine-mediated DNA-PK stimulation on HIV transcription and replication, we treated the Jurkat T cells (Figure 5B,C) and PBMCs (Figure 5D,E) with M3814 overnight (24 h). The next day, fresh media was provided with cocaine for 3 h. After 3 h of cocaine exposure, the cells were infected with a competent replication virus (93/TH/051) for 24 h. The HIV transcripts were quantified using real-time qPCR, using primer sets that amplify the Nuc-2 (Figure 5B,D) and Env (Figure 5C,E) regions of the HIV genome. A significant upregulation of the HIV transcript was observed in the presence of cocaine, but, as anticipated, the presence of M3814 strongly restricted HIV gene expression in a dose-dependent manner (Figure 5B–E). These results were further validated by examining the expression of the HIV protein in the absence or presence of M3814. The Jurkat cells were treated with M3814 for 24 h. The next day, the cells were treated with cocaine for 3 h. Later, we infected the cells with replication-competent HIV (93/TH/051) for another 24 h. The cell lysates were then subjected to immunoblotting to detect HIV proteins p24 and p17. Again, we noted higher levels of HIV proteins p24 and p17 following cocaine exposure. However, in the presence of an inhibitor, the level of HIV proteins dropped sharply, further demonstrating that the cocaine-induced DNA-PK plays a crucial role in HIV transcription and replication (Figure 5F,G). Overall, these results confirm that cocaine-mediated DNA-PK stimulation is required for HIV gene expression and consequently for HIV replication.

### 3.5. Cocaine Promotes HIV Transcription by Enhancing the Phosphorylation of the C-Terminal Domain (CTD) of RNA Polymerase II (RNAP II)

**Figure 6:**
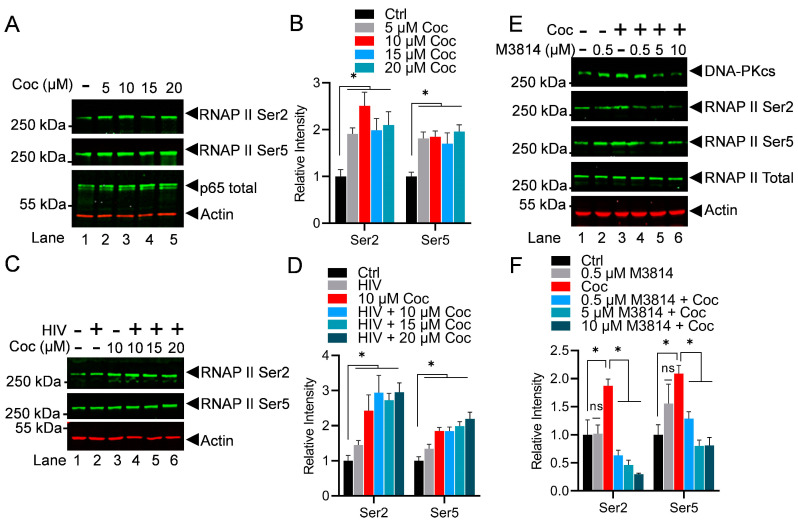
Cocaine promotes HIV transcription by enhancing the phosphorylation of the C-terminal domain (CTD) of RNA polymerase II (RNAP II). THP-1 cells were treated with increasing doses of cocaine (5, 10, 15, and 20 µM) for 3 h (**A**). MT-4 cells were treated as follows: untreated and uninfected (Lane 1), infected with HIV (93/TH/051) without cocaine treatment (Lane 2), treated with cocaine without HIV infection (Lane 3), or pre-treated with different concentrations of cocaine before HIV infection (Lanes 4 to 6) (**C**). Cells were harvested, and nuclear lysates were analyzed by immunoblotting with specific antibodies against phosphorylated RNAP II, RNAP II Ser2, and RNAP II Ser5. Actin, a constitutively expressed protein, was used as the loading control. Densitometric analysis of protein bands (normalized to actin) confirmed the significant hyper-phosphorylation of the RNAP II CTD within both Ser2 and Ser5 residues following cocaine treatment (**B**,**D**). THP-1 cells were treated with cocaine in the absence or presence of different concentrations of M3814 (0.5, 5, and 10 µM) (**E**). Cells were harvested, and nuclear extracts were evaluated via immunoblotting using specific antibodies against RNAP II Ser2, RNAP II Ser5, and total RNAP II. Densitometric analysis of protein bands (normalized to actin) (**F**). Immunoblots are representative of at least three independent experiments. The results are expressed as the mean ± SD and analyzed by two-way ANOVA followed by Tukey’s multiple comparison test. “ns” indicates not significant. Asterisks over the bars indicate significant differences. * *p* < 0.05 is for the comparison of cocaine-treated samples against untreated (Ctrl) and the comparison of cocaine plus inhibitors treated against cocaine-alone-treated samples.

RNAP II is the main enzyme that transcribes eukaryotic DNA into mRNA. The C-terminal domain (CTD) of RNAP II consists of a repeating sequence of seven amino acids (heptapeptide) with the consensus sequence Tyr1-Ser2-Pro3-Thr4-Ser5-Pro6-Ser7 (YSPTSPS) around 52 times [32,33,34,35]. All residues within the CTD heptad repeat can be post-translationally modified by phosphorylation (tyrosine, threonine, serine, and proline). However, in the RNAP II CTD, serine 5 and serine 2 phosphorylation (Ser5-P and Ser2-P) are the best-studied and the most established indicators of ongoing transcription. Specifically, the phosphorylation of the RNAP II CTD at Ser5 is linked to the initiation phase of transcription, marking the initial movement of RNAP II from the gene promoter, whereas phosphorylation of Ser2 is found to be correlated with the elongation phase of transcription. Notably, to generate a full-length HIV transcript, both initiation and elongation phases are required. Therefore, we evaluated if cocaine enhances HIV transcription by hyper-phosphorylating the RNAP II CTD, and we analyzed the phosphorylation of the RNAP II CTD at Ser2 and Ser5 upon cocaine exposure. THP-1 cells were treated with increasing concentrations of cocaine for 3 h. Later, the nuclear lysate was subjected to immunoblotting to probe with RNAP II Ser2-P, RNAP II Ser5-P, and RNAP II total. The activation of p65, a subunit of NF-kB, was analyzed as a positive control to confirm cocaine-mediated cell stimulation. As anticipated, we observed the stimulation of p65, marked by an enhanced level of p65 in the nucleus compared to untreated cells (Ctrl). Notably, we also found hyper-phosphorylates of the RNAP II CTD within both the Ser2 and Ser5 residues following cocaine treatment (Figure 6A,B). The dose-dependent upregulation of RNAP II CTD phosphorylation further confirmed the direct impact of cocaine in enhancing the phosphorylation of the RNAP II CTD.

To further validate the ubiquity of our findings, the results were confirmed in MT-4 cells. The cells were treated with different doses of cocaine for 3 h before being infected with dual tropic HIV (93/TH/051). After 3 h, the nuclear extracts were examined for RNAP II at Ser2 and Ser5. The hyper-phosphorylation of RNAP II at both the Ser2 and Ser5 positions of the RNAP II CTD upon cocaine treatment was evaluated (Figure 6C,D). The dose-dependent hyper-phosphorylation of the RNAP II CTD was clearly evident.

Subsequently, we examined if the DNA-PK is involved in the cocaine-induced RNAP II CTD phosphorylation. We hypothesized that if the cocaine-induced DNA-PK catalyzes RNAP II CTD phosphorylation, then the inhibition of the DNA-PK should impair cocaine stimulated RNAP II CTD hyper-phosphorylation. To test this hypothesis, the THP-1 cells were treated with increasing concentrations of M3814 for 24 h. The next day, the cells were exposed to cocaine for 3 h. Later, nuclear protein lysates were analyzed by immunoblotting to examine RNAP II CTD phosphorylation at the sites Ser2 and Ser5. As shown in the figure (Figure 6E), cocaine treatment significantly upregulated RNAP II CTD phosphorylation at Ser2 and Ser5, validating the above results. We noted a significant reduction in CTD phosphorylation at both Ser2 and Ser5 in the presence of M3814 when compared to the cocaine-alone samples. The dose-dependent inhibition of RNAP II CTD phosphorylation at both Ser5 and Ser2 with M3814 confirmed our hypothesis and validated that the cocaine-stimulated DNA-PK plays a vital role in promoting both the initiation and elongation phases of HIV transcription by catalyzing both Ser5 and Ser 2, respectively (Figure 6E,F). Overall, the results demonstrate that by activating the DNA-PK, cocaine promotes different stages of HIV transcription, a necessity to produce complete HIV genomic transcripts or new HIV progeny.

### 3.6. Cocaine Enhances the Elongation Phase of HIV Transcription Not Only by Stimulating the DNA-PK but Also via P-TEFb Activation

**Figure 7:**
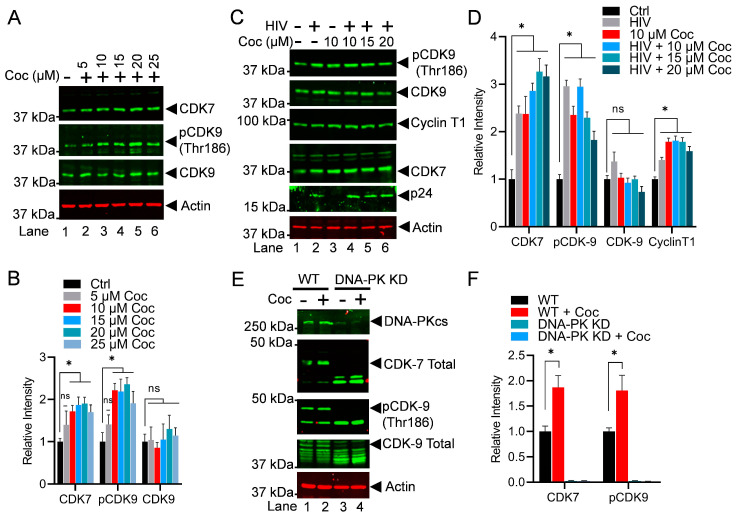
Cocaine enhances the elongation phase of HIV transcription, not only by stimulating the DNA-PK, but also P-TEFb. Jurkat-pHR’P-Luc cells were treated with increasing doses of cocaine (5, 10, 15, 20, and 25 µM) for 3 h (**A**). Jurkat-pHR’P-Luc cells were treated as follows: untreated and uninfected (Lane 1), infected with HIV (93/TH/051) without cocaine treatment (Lane 2), treated with cocaine without HIV infection (Lane 3), or pre-treated with different concentrations of cocaine before HIV infection (Lanes 4 to 6) (**C**). Cells were harvested, and nuclear lysates were analyzed by immunoblotting with specific antibodies against P-TEFb subunits CDK9 and Cyclin T1, as well as CDK7 (TFIIH). Actin was used as a loading control. Densitometric analysis of protein bands (normalized to actin) confirmed a significant increase in CDK7, Cyclin T1, and p-CDK9 (Thr186) compared to untreated (Ctrl) cells (**B**,**D**). Wild-type (WT) and DNA-PK knockdown (DNA-PK KD) cells were treated with cocaine for 30 min and 3 h, and nuclear extracts were subjected to immunoblotting (**E**). Densitometric analysis of protein bands (normalized to actin) showed increased p-CDK9 phosphorylation and CDK7 activation in WT cells upon cocaine exposure (**F**). However, in DNA-PK KD cells, the lack of p-CDK9 (Thr186) phosphorylation and CDK7 activation upon cocaine treatment demonstrated that cocaine-induced activations are DNA-PK-specific (**F**). Immunoblots are representative of at least three independent experiments. The results are expressed as the mean ± SD for three independent experiments, analyzed by two-way ANOVA followed by Tukey’s multiple comparisons test. “ns” indicates not significant. Asterisks over the bars indicate significant differences: * *p* < 0.05 compared to untreated cells (Ctrl).

The above results demonstrate that cocaine promotes both the initiation and elongation phases of HIV transcription by enhancing RNAP II CTD phosphorylation at Ser5 and Ser2. We further investigated if cocaine promotes the elongation phase by stimulating P-TEFb. CDK9 is the kinase subunit of the P-TEFb complex, which plays a crucial role in catalyzing the phosphorylation of the RNAP II CTD at position Ser2, a post-translational modification that makes RNAP II processive or elongation-proficient. We examined the stimulation of P-TEFb following cocaine exposure. Jurkat-pHR’P-Luc cells were exposed to increasing doses of cocaine for 3 h. Subsequently, the nuclear lysates were subjected to immunoblotting using specific antibodies against CDK7 (TFIIH), p-CDK9 (thr186), and total CDK9. The data indicated that cocaine enhances CDK7, thereby facilitating the initiation of HIV transcription. Additionally, the data show an increase in the phosphorylation of CDK9 at threonine residue 186, which marks functionally active CDK9. However, cocaine did not affect the level of total CDK9 (Figure 7A,B). To further validate these findings, Jurkat-pHR’P-Luc cells were treated with escalating doses of cocaine for 2 h and subsequently infected with replication-competent HIV (strain 93/TH/051) for an additional hour. As shown in Figure 7C, Jurkat-pHR’P-Luc cells were treated as follows: untreated and uninfected (Lane 1), infected with HIV (93/TH/051) in the absence of cocaine (Lane 2), treated with cocaine without HIV infection (Lane 3), or pre-treatment with different concentrations of cocaine before infecting with HIV (Lane 4–6). The nuclear lysates were analyzed via immunoblotting using specific antibodies against the main P-TEFb subunits, CDK9 and Cyclin T1. The obtained data clearly show the enhanced phosphorylation of CDK9 and also the upregulation of Cyclin T1 upon cocaine treatment, demonstrating that cocaine further supports the ongoing elongation phase of HIV transcription by stimulating CDK9. Nevertheless, it does not affect the level of CDK9. Actin was used as a loading control, while p24 was probed to mark ongoing HIV replication. Densitometric analysis of protein bands validated a significant increase in p-CDK9 (thr186) and Cyclin T1 but not CDK9 total levels compared to untreated cells (control) (Figure 7C,D). We also evaluated the impact of cocaine on another kinase, CDK7, which is a component of the TFIIH complex that is mainly responsible for Ser5 phosphorylation, another RNAP II CTD modification required for the initiation phase of transcription. As expected, we noted the upregulation of CDK7 upon cocaine treatment. The results again demonstrate that by enhancing CDK7, cocaine facilitates the initiation phase of HIV transcription.

To further substantiate that the cocaine-induced phosphorylation of CDK9 and the activation of CDK7 are due to DNA-PK activation, we conducted experiments using DNA-PK knockdown cells. Wild-type (WT) and DNA-PK knockdown (DNA-PK KD) cells were treated with cocaine for 3 h. Subsequently, we analyzed the phosphorylation of CDK9 and the activation of CDK7. We found a significant reduction in p-CDK9 (Thr186) levels, as well as in total CDK9 and CDK7 following DNA-PK depletion (Figure 7E,F). Our findings in wild-type cells confirmed our above results, indicating that cocaine exposure led to an increase in pCDK9 phosphorylation and the activation of CDK7. However, in DNA-PK KD cells, we observed a persistent reduction in pCDK9 (thr186) phosphorylation and CDK7 activation upon cocaine exposure, suggesting that cocaine-induced CDK9 phosphorylation and the activation of CDK7 are DNA-PK-specific. Together, these findings confirmed our hypothesis that the cocaine-induced DNA-PK facilitates the initiation and elongation phases of HIV transcription by stimulating CDK7 (TFIIH) and CDK9 (P-TEFb), respectively.

### 3.7. Cocaine-Induced DNA-PK Relieves RNAP II Pausing by Phosphorylating TRIM28 at S824

**Figure 8:**
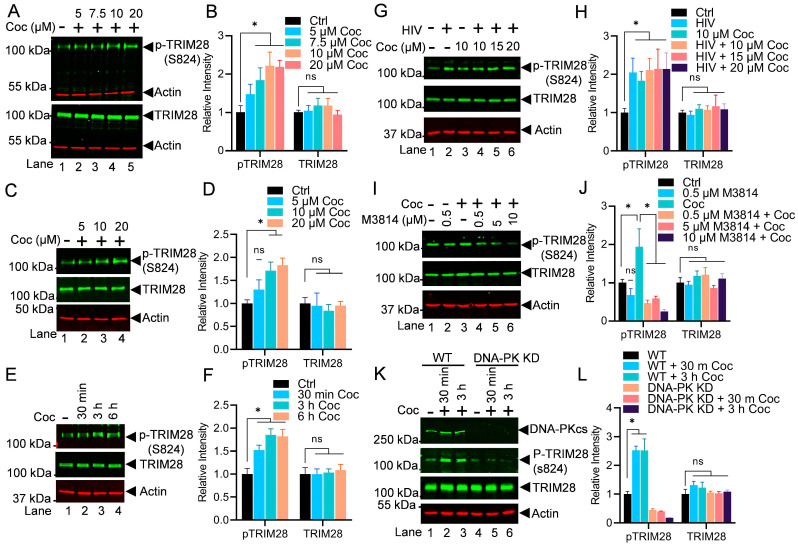
The cocaine-induced DNA-PK relieves RNAP II pausing by phosphorylating TRIM28 at S824. THP-1 (**A**,**B**) and Jurkat cells (**C**,**D**) were treated with increasing doses of cocaine, and the nuclear lysates were quantified and then evaluated via immunoblotting using specific antibodies against pTRIM28 (S824) and total TRIM28. Densitometric analysis confirmed a significant increase in pTRIM28 (S824) levels compared to untreated cells (Ctrl) (**A**–**D**). Jurkat-pHR’P-Luc cells were treated with cocaine (10 µM) for varying durations (30 min, 3 h, and 6 h), and the nuclear lysates were analyzed via immunoblotting using specific antibodies against pTRIM28 (S824) and total TRIM28. Densitometric analysis of protein bands (normalized to actin) confirmed a significant increase in pTRIM28 (S824) levels compared to untreated cells (Ctrl) (**E**,**F**). THP-1 cells were treated as follows: untreated and uninfected (Lane 1), infected with HIV (93/TH/051) without cocaine (Lane 2), treated with cocaine without HIV infection (Lane 3), or pre-treated with different concentrations of cocaine before HIV infection (Lanes 4 to 6). Nuclear lysates were analyzed via immunoblotting using specific antibodies against pTRIM28 (S824) and total TRIM28 (**G**). Densitometric analysis of protein bands (normalized to actin) confirmed a significant increase in pTRIM28 (S824) levels compared to untreated cells (Ctrl) (**H**). THP-1 cells were treated with different concentrations of M3814 in the presence and absence of cocaine (10 µM), and the nuclear lysates were analyzed via immunoblotting using specific antibodies against pTRIM28 (S824) and total TRIM28 (**I**). Densitometric analysis of protein bands (normalized to actin) (**J**). WT and DNA-PK KD cells were treated with cocaine for 30 min and 3 h, and the nuclear lysates were subjected to immunoblotting (**K**). Densitometric analysis of protein bands (normalized to actin) (**L**). Immunoblots are representative of at least three independent experiments. The results are expressed as the mean ± SD for three independent experiments, analyzed by two-way ANOVA followed by Tukey’s multiple comparisons test. “ns” indicates not significant. Asterisks over the bars indicate significant differences. * *p* < 0.05 is for the comparison of cocaine-treated samples against untreated (Ctrl) and the comparison of cocaine plus inhibitors treated against cocaine alone-treated samples.

Later, we examined the impact of cocaine-induced TRIM28 activation (p-TRIM28-(S824) in relieving RNAP II pausing. TRIM28 is one of the RNAP II pausing factors, which restricts the flow of RNAP II on the DNA template after transcription of the first 50 to 60 nucleotides. Additionally, it has been recently documented that TRIM28 potently suppresses HIV expression by utilizing both SUMO E3 ligase activity and epigenetic adaptor function [39,68,71]. However, the phosphorylation of TRIM28 at its Ser824 converts TRIM28 from a pausing factor to a transcription-supporting factor [39,59,60]. To further extend our previous findings [39], in this investigation, for the first time, we provide the evidence that the DNA-PK is the main kinase that catalyzes the phosphorylation of TRIM28 at Ser824 (p-TRIM28-(S824) and reverses the inhibitory effect of TRIM28 on HIV gene transcription. We hypothesized that if cocaine stimulates the DNA-PK and plays a major role in supporting not only initiation but also the elongation phase of HIV transcription, then the cocaine-induced DNA-PK should be able to relieve RNAP II pausing, a prerequisite for the elongation phase of transcription. To test this hypothesis, we examined the neutralization of RNAP II pausing through the conversion of TRIM28 from a transcriptionally repressive factor (TRIM28) to a transcriptionally active factor (p-TRIM28 S824) by the cocaine-induced DNA-PK-mediated phosphorylation of TRIM28. The THP-1 cells, a myeloid cell line, were treated with increasing doses of cocaine for 3 h. The nuclear lysates were analyzed by immunoblotting to detect the phosphorylated form of TRIM28 (p-TRIM28-(S824) and total TRIM28. The expression of the actin protein among samples was evaluated as the loading control. As expected, following cocaine exposure, we found enhanced TRIM28 phosphorylation at the position S824 (p-TRIM28-(S824) in a dose-dependent manner (Figure 8A,B). The densitometric analyses of protein bands further establish the significant dose-dependent increase in p-TRIM28-(S824) levels upon cocaine treatment compared to the untreated cell control. Thus, this shows that cocaine, by enhancing phosphorylation of TRIM28, relieves RNAP II pausing during HIV transcription. These results were further confirmed in Jurkat cells, a lymphoid cell line (Figure 8C,D).

To examine the kinetics of TRIM28 phosphorylation upon cocaine exposure, we treated the Jurkat-pHR’P-Luc cells with a fixed dose of cocaine (10 μM) for different durations: 30 min, 3 h, and 6 h (Figure 8E,F). Then, we analyzed the nuclear lysates to assess the levels of p-TRIM28 (S824) and TRIM28 total; we noted significant phosphorylation of TRIM28 at 3 h upon cocaine exposure. As anticipated, densitometry analyses revealed a significant increase in TRIM28 phosphorylation following cocaine treatment, displaying a distinct kinetic profile (Figure 8E,F). Together, our data establish that cocaine-mediated enhanced TRIM28 phosphorylation (p-TRIM28-(S824) plays a crucial role in transitioning HIV transcription from pausing to the elongating phase by antagonizing the pausing effect of TRIM28, and thus, promoting RNAP II pause release.

The results were also reproduced in Jurkat cells infected with replication-competent virus (93/TH/051). Jurkat-pHR’P-Luc cells were treated with increasing concentrations of cocaine for 3 h before being infected with 93/TH/051. After 3 h, the nuclear extracts were examined for p-TRIM28 (S824) and TRIM28 total. The enhanced phosphorylation of TRIM28 at S824 (p-TRIM28-(S824) upon cocaine treatment was confirmed (Figure 8G,H).

Subsequently, to determine if the cocaine-induced DNA-PK is responsible for TRIM28 phosphorylation (p-TRIM28 S824), we examined the impact of DNA-PK inhibition on TRIM28 phosphorylation. We found a dose-dependent inhibition of TRIM28 phosphorylation and an almost complete elimination of TRIM28 phosphorylation (p-TRIM28 S824) in cells treated with 10 µM M3814 (Figure 8I,J). Together, these findings confirm that the cocaine-induced DNA-PK plays a vital role in RNAP II pause release by enhancing TRIM28 phosphorylation at a specific site (p-TRIM28-(S824), which converts TRIM28 from an inhibitory factor to a transactivator (Figure 8).

We further confirmed the specific role of the cocaine-stimulated DNA-PK in catalyzing the phosphorylation of TRIM28 and reversing its inhibitory effect during HIV transcription by performing experiments using DNA-PK knockdown (KD) cells. Cells were infected with lentiviral vectors expressing shRNA either against the catalytic subunit of DNA-PK (DNA-PKcs) or the scrambled shRNA that does not target any cellular gene. These cells were treated with cocaine for 30 min and 3 h. Later, the phosphorylation of p-TRIM28 at S824 and total TRIM28 was analyzed. In DNA-PK knockdown cells, we observed a clear reduction in the levels of p-TRIM28-(S824) but not TRIM28 (Figure 8K,L). However, in cells harboring scrambled shRNA, which express normal levels of the DNA-PK, we noted an enhanced phosphorylation level of p-TRIM28 upon the cocaine exposure, validating our previous results. We also noted the level of phosphorylated TRIM28 remains reduced in the DNA-PK KD cells upon exposure to cocaine, confirming that cocaine-induced TRIM28 phosphorylation is DNA-PK-specific. Thus, the results demonstrated that the enhanced phosphorylation of TRIM28 induced by cocaine is directly associated with the stimulation of DNA-PK triggered by cocaine (Figure 8K).

**Figure 9:**
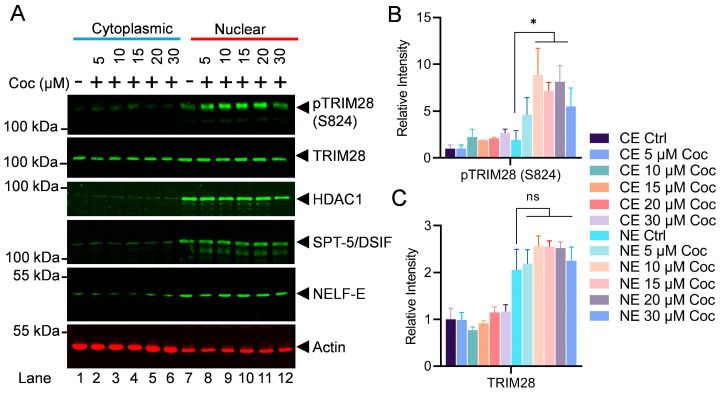
Cocaine promotes RNAP II pause release by phosphorylating TRIM28 at S824. Jurkat cells were exposed to increasing concentrations of cocaine, and both cytoplasmic and nuclear extracts were subjected to immunoblotting using specific antibodies against pTRIM28 (S824), total TRIM28, DSIF (SPT-5), NELF-E, and HDAC1 (**A**). Densitometric analysis of protein bands (normalized to actin) (**B**,**C**). Immunoblots are representative of at least three independent experiments. The results are expressed as the mean ± SD for three independent experiments, analyzed by two-way ANOVA followed by Tukey’s multiple comparisons test. “ns” indicates not significant. Asterisks over the bars indicate significant differences. Statistical significance is set as *p* < 0.05 (*) compared to untreated cells (Ctrl).

To understand the cellular kinetics of TRIM28, we analyzed the cytosolic and nuclear levels of p-TRIM28 (S824) and TRIM28 upon cocaine exposure. We also analyzed the impact of cocaine on two main RNAP II pausing factors, namely the DSIF (SPT-5) and the NELF (NELF-E). Interestingly, we did not observe any significant changes in the DSIF and the NELF upon cocaine exposure (Figure 9A–C). These results clearly document that cocaine primarily relieves RNAP II pausing by inducing the DNA-PK-mediated phosphorylation of TRIM28 (p-TRIM28-(S824). Altogether, our data validate that the cocaine-stimulated DNA-PK relieves RNAP II pausing by antagonizing the effect of negative/pausing factors, mainly TRIM28, via its phosphorylation at ser824 (p-TRIM28-(S824), during HIV transcription.

### 3.8. Cocaine Boosts HIV Transcription by Enhancing the Recruitment of the DNA-PK and pTRIM28 at the HIV LTR

**Figure 10:**
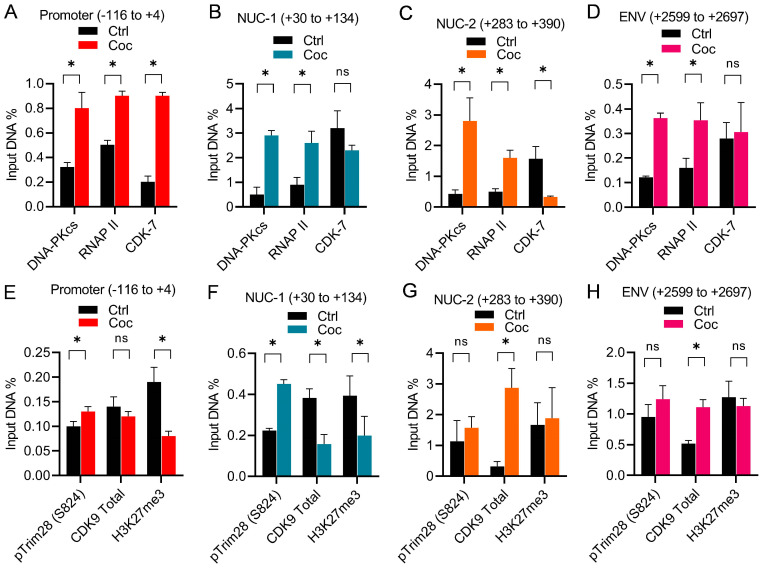
Cocaine enhances HIV transcription by promoting the recruitment of the DNA-PKcs and pTRIM28 (S824) at the HIV LTR. Jurkat cells freshly infected with replication-incompetent HIV and pHR’P-Luc were exposed to cocaine. Chromatin immunoprecipitation (ChIP) assays were conducted to assess the recruitment kinetics of the DNA-PKcs, RNAP II, CDK7 (TFIIH), pTRIM28 (S824), total CDK9, and H3K27me3 at the promoter (**A**,**E**), Nucleosome-1 (**B**,**F**), Nucleosome-2 (**C**,**G**), and the further downstream Envelope regions (**D**,**H**) of the HIV LTR, using specific primer sets. The results are presented as the mean ± SD for three independent experiments, analyzed by two-way ANOVA followed by Tukey’s multiple comparisons test. “ns” indicates not significant. Asterisks above the bars indicate significant differences. Statistical significance is set as *p* < 0.05 (*) compared to untreated cells (Ctrl).

Previously, we documented the parallel presence of the DNA-PK along with RNAP II throughout the HIV proviral genome during HIV transcription [37,39]. Additionally, we showed the recruitment of TRIM28 at the HIV long terminal repeat (LTR) during HIV transcription [39]. We also found that cell activation enhances both the nuclear localization and LTR recruitment of the DNA-PK [39]. Given that cocaine further augments the nuclear level of the DNA-PK, we hypothesize that the enhanced nuclear localization of the DNA-PK should be translated into the higher recruitment of the DNA-PK and pTRIM28 at the HIV LTR. To test this hypothesis, we evaluated the recruitment of the DNA-PK and p-TRIM28-(S824) at the HIV LTR in the presence and absence of cocaine by chromatin immunoprecipitation (ChIP) assay using our standard methodology [28,37,75,85,99]. The ChIP assays were performed using antibodies against the DNA-PKcs, RNAP II, CDK7, CDK9, p-TRIM28 (S824), and H3K27me3. The analysis was conducted in Jurkat cells freshly infected with p-HR’P-Luc (Figure 2A). The recruitment of RNAP II at the HIV LTR was assessed as a positive control to mark the status of ongoing HIV transcription. We examined CDK7 as a marker of transcriptional initiation, as CDK7 (TFIIH) plays a role during the initiation phase of HIV transcription. The recruitment of CDK9 (P-TEFb) at the HIV LTR was evaluated to indicate the elongation phase of HIV transcription, as the recruitment of P-TEFb is crucial to support HIV transcriptional elongation. The immunoprecipitated DNA was analyzed using four primer sets targeting different regions of the HIV LTR. The first primer set amplifies the promoter region of the LTR (−116 to +4 with respect to the transcription start site, Figure 10A,E). The second primer set amplifies the Nuc-1 region of the LTR (+30 to +134 with respect to the transcription start site, Figure 10B,F). The factors that mainly bind at the promoter and Nuc-1 region usually mark factors involved in the initiation phase of HIV transcription. The third primer set amplifies the downstream Nuc-2 region of the LTR (+283 to +390 with respect to the transcription start site, Figure 10C,G). The fourth primer set amplifies the further downstream ENV region of HIV (+2599 to +2697, Figure 10D,H). The factors that bind around the Nuc-2 region and downstream from it primarily represent those involved in the elongation phase of transcription. Following cocaine treatment, as anticipated, we found higher recruitment of RNAP II, showing upregulation of HIV transcription. Moreover, enhanced RNAP II levels at the promoter, Nuc-1, Nuc-2, and Env regions of provirus in cocaine-treated cells indicate enhanced ongoing HIV gene expression upon cocaine treatment. Interestingly, in parallel to the recruitment of RNAP II, we observed the significantly enhanced recruitment of the DNA-PKcs at the promoter, Nuc-1, Nuc-2, and the Env regions of the LTR following cocaine treatment (Figure 10A–D). These results corroborate our previous data, where we showed the continuous presence and gliding of the DNA-PKcs with RNAP II along the HIV genome during transcription [37]. Notably, we also found the enrichment of p-TRIM28-(S824) at the promoter and Nuc-1 regions (Figure 10E,F). However, we did not observe significant changes in the Nuc-2 and Env regions of the HIV LTR (Figure 10G,H). Meanwhile, we noted exclusively higher recruitment of CDK7 (kinase subunit of TFIIH) at the promoter of the LTR region (Figure 10A–D). The finding that after cocaine stimulation, CDK7 was enriched at the LTR promoter but not at the downstream regions, validates its involvement, specifically during the initiation phase of HIV transcription. Interestingly, the loss of H3K27Me3 from the HIV LTR following cocaine treatment demonstrates the removal of transcriptionally repressive (heterochromatin) structure and the establishment of a transcriptionally active (euchromatin) structure at the HIV LTR following cocaine treatment. These data further validate our previous findings, where we showed that cocaine enhances HIV transcription by promoting the euchromatin structure at the HIV LTR [28]. As anticipated, following cocaine exposure, we also found enhanced recruitment of CDK9 (kinase subunit of P-TEFB), specifically at the downstream region of LTR but not much at the promoter region, validating its role during the elongation phase of transcription (Figure 10G,H). Following cocaine exposure, the specific enrichment of CDK9 (P-TEFb) at the downstream region of the LTR and CDK7 (TFIIH) at the promoter region validates the authenticity of our assay system and ChIP analysis.

Overall, our results demonstrate that cocaine stimulates and enhances the nuclear localization and catalytic activity of the DNA-PK (p-DNA-PK S2056), which leads to its higher recruitment at the HIV LTR. The DNA-PK subsequently catalyzes the phosphorylation of TRIM28 (p-TRIM28 S824) and converts TRIM28 from a pausing factor to a transcription activator. Overall, these modifications relieve RNAP II pausing and promote HIV transcriptional elongation, a necessity to make complete HIV genomic transcripts, which are required for generating viral progeny.

### 3.9. Cocaine-Induced DNA-PK Activation Promotes HIV Transcription by Supporting Several Aspects of HIV Transcription

**Figure 11:**
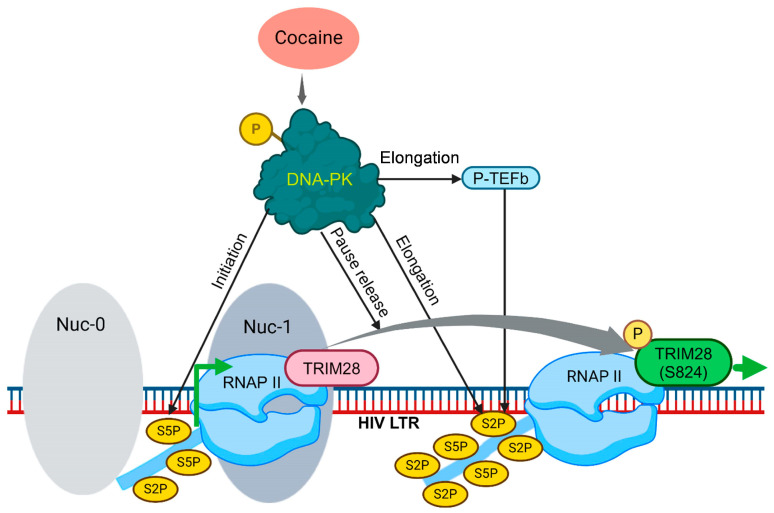
Cocaine-mediated DNA-PK activation enhances multiple aspects of HIV transcription. Cocaine-induced DNA-PK activation facilitates various stages of HIV transcription. Firstly, it enhances the initiation phase of HIV transcription by phosphorylating the C-terminal domain (CTD) of RNA polymerase II (RNAP II) at Ser5. Secondly, the cocaine-stimulated DNA-PK promotes the elongation phase by both directly catalyzing and facilitating the recruitment of positive transcription elongation factor b (P-TEFb), leading to the phosphorylation of Ser2 within the RNAP II CTD. This posttranslational modification renders RNAP II processive, ensuring efficient elongation. Finally, cocaine-induced DNA-PK activity also alleviates RNAP II pausing by phosphorylating TRIM28 at Ser824 (p-TRIM28 S824). This modification transforms TRIM28 from a transcriptional pausing factor to a facilitator (transactivator), thereby supporting HIV transcription.

To summarize our findings from current and previous investigations, we present the following model for the DNA-PK’s role during HIV transcription (Figure 11) [37,39]. In our previous studies, we established the association of the DNA-PK and RNAP II along the HIV proviral DNA template throughout HIV gene expression. In this study, we found that cocaine exposure augments the nuclear localization and functional activation of the DNA-PK (p-DNA-PK S2056). The DNA-PK subsequently facilitates the multiple critical phases of HIV transcription, namely initiation, RNAP II pause release, and elongation. The cocaine-induced DNA-PK promotes the initiation phase of transcription by catalyzing the phosphorylation of the RNAP II CTD at Ser5. In addition, the cocaine-stimulated DNA-PK facilitates the elongation phase of HIV transcription by both directly catalyzing and promoting the recruitment of P-TEFb for the phosphorylation of Ser2 within the RNAP II CTD. The hyperphosphorylation of the RNAP II CTD at Ser2 makes RNAP II processive or elongation-proficient. Another noteworthy finding is that the cocaine-stimulated DNA-PK relieves RNAP II pausing selectively through TRIM28 by catalyzing TRIM28 phosphorylation at Ser824 (p-TRIM28 S824). This modification transforms TRIM28 from a transcription-pausing factor to a transcription-supporting factor. Thus, the phosphorylation of TRIM28 at Ser824 relieves RNAP II pausing and allows RNAP II to proceed along the DNA template or transcriptional elongation. Our findings collectively underscore the profound impact of cocaine-induced DNA-PK activation on various facets of HIV transcription, ultimately culminating in the potent promotion of viral gene expression.

## 4. Discussion

HIV/AIDS remain a dreadful disease, as an effective vaccine or cure is yet to be developed [5,100,101,102,103,104]. Nevertheless, with the introduction of ART, the life quality of PLWH significantly increases [1,6]. However, one has to rely on medication for the rest of one’s life to keep control of HIV disease progression. The anti-HIV therapy is highly effective in suppressing viral replication, maintaining a healthy immune system, and reducing the risk of HIV transmission. Unfortunately, cocaine, one of the drugs most abused by HIV patients, can disrupt regular activities, potentially leading to inconsistent or missed doses of ART. Poor adherence usually leads to treatment failure, the development of a drug-resistant HIV strain, and compromised immune functions [105,106]. Cocaine further affects the normal functioning of immune cells, suppressing the immune system and exacerbating the effect of HIV infection, leading to faster disease progression, and making HIV patients especially vulnerable to opportunistic infections. Furthermore, given that cocaine strongly impacts brain functioning, cocaine use by HIV patients not only accelerates HIV replication in the CNS but also exacerbates normal brain functioning. The interaction between cocaine and HIV is a multifaceted and concerning issue. Therefore, understanding the molecular mechanisms that govern the HIV life cycle, especially transcription and replication, is crucial for relieving HIV and cocaine-induced neurotoxicity in addition to HIV cure and eradication [16,17,27,39].

In this study, we showed the pivotal role played by the cocaine-induced activation of the DNA-PK in bolstering various stages of HIV transcription, consequently augmenting HIV replication. Our investigation has unveiled that cocaine significantly upregulates the expression of the DNA-PK, prompts its localization into the nucleus, and enhances the functional activity of the DNA-PK by enhancing its phosphorylation at S2056. Subsequently, the cocaine-induced DNA-PK facilitates transcriptional initiation by augmenting the phosphorylation of the CTD at Ser5, relieves RNAP II pausing through TRIM28 phosphorylation at S824, and promotes transcriptional elongation both by directly catalyzing the phosphorylation of the CTD at Ser2 and through P-TEFb stimulation and recruitment. Accordingly, upon specific inhibition or depletion of the DNA-PK using specific inhibitors or knockdown, respectively, we found profound restrictions to cocaine-induced HIV transcription and replication. These collective results unveil the underlying molecular mechanisms through which cocaine-induced DNA-PK stimulation augments HIV transcription and replication.

The DNA-PK is a serine/threonine protein kinase complex composed of a heterodimer of Ku proteins (Ku70/Ku80) and a catalytic subunit, DNA-PKcs [40,41]. The DNA-PK is a critical component of the cellular response following DNA damage [40,41]. The DNA-PK is one of the main components of the DNA repair pathway upon double-strand breaks, especially in the NHEJ DNA double-strand break repair pathway [43,44]. Therefore, the DNA-PK is extensively studied in DNA double-strand break repair. The role of the DNA-PK in HIV transcription was first identified as a complex that phosphorylates the transcription factor SP1 [107] and as an interacting component of RNAP II [47]. Nevertheless, its role in transcription has been understudied. For the first time, we demonstrated the precise role of the DNA-PK during any transcription process by defining the mechanism through which the DNA-PK promotes HIV transcription and the involved mechanisms [37,39]. Later, several studies emerged that further strengthened the link between the DNA-PK and transcriptional regulation [108]. HIV transcription is a fundamental step that plays a crucial role in regulating HIV replication and latency-reactivation. In our previous studies, we have documented the underlying molecular mechanism through which the DNA-PK promotes HIV transcription [39]. Moreover, we found that cocaine also enhances HIV transcription and replication [16,27,28]. These facts prompted us to study whether cocaine-enhanced HIV transcription and replication is due to the activation of the DNA-PK. In this investigation, we demonstrated that cocaine significantly upregulates the nuclear level of the DNA-PK and augments its activity by enhancing its phosphorylation at serine 2056 residues. We reproduced these findings in cells of different lineages, including both lymphoid and myeloid lineages. Given that in our previous findings, we noted the higher recruitment of the DNA-PK at the HIV LTR following cell stimulation [37,39], we evaluated if cell stimulation by cocaine also results in the enhanced nuclear localization of the DNA-PK. We found that cocaine-induced cell stimulation was sufficient to increase the nuclear levels of both the total DNA-PK and also the phosphorylated DNA-PK (p-DNA-PK S2056) (Figure 1). The enhanced nuclear localization of the DNA-PK upon cocaine exposure was analyzed via both immunofluorescence and immunoblotting by comparing cocaine-treated cells with untreated cell control. As anticipated, we noted significantly increased levels of the DNA-PK and p-DNA-PK (S2056) in the nucleus following cocaine treatment (Figure 1G–I). Subsequently, we analyzed the corresponding recruitment of the DNA-PK at the HIV LTR due to the higher availability of the DNA-PK in the nucleus by ChIP assays. As expected, upon cocaine exposure, we found a notable increase in the recruitment of the DNA-PK. Additionally, along with the DNA-PK, we found the corresponding higher recruitment of RNAP II at the HIV LTR following cocaine treatment (Figure 10A–D). This finding reaffirmed our prior findings where we established DNA-PK interaction with RNAP II and showed parallel-enhanced recruitment of both the DNA-PK and RNAP II following cell stimulation [37]. Interestingly, paralleling the recruitment of RNAP II, we also noted the augmented recruitment of the DNA-PK, not only at the promoter and Nuc-1 regions, but also at the downstream regions of the HIV genome (Figure 10A–D). These findings argued for the role of the DNA-PK in different phases of HIV transcription, including initiation, RNAP II pause release, and elongation phases. Accordingly, we found higher levels of RNAP II at the promoter and Nuc-1, the region of the provirus, signifying enhanced ongoing HIV gene expression following cocaine exposure. This observation further strengthens our previous results proposing that the DNA-PK and RNAP II are part of a larger transcription complex [37,39]. Later, we assessed if HIV infection promotes cell stimulation and consequently DNA-PK activation. Notably, we found a significant upregulation of the DNA-PK and its activation (p-DNA-PK S2056) upon cocaine exposure, documenting a crucial role of the DNA-PK during HIV transcription. Together, these findings underscore the intricate relationship between cocaine exposure and the LTR recruitment of the DNA-PK, shedding light on the potential mechanism through which cocaine augments HIV transcription.

TRIM28 is a multifaceted protein that is involved in a wide range of cellular processes, including the regulation of gene expression, cell growth and differentiation, pluripotency, neoplastic transformation, apoptosis, DNA repair, and the preservation of genomic integrity. As a key partner of the main kinases, the DNA-PK and ATM, of DNA repair machinery, TRIM28 plays a vital role in the DNA repair process [109,110]. The rapid recruitment of TRIM28 along with Heterochromatin Protein 1 (HP-1) to the sites of DNA double-strand break is well documented [58,109,111]. Initially, SUMOylated TRIM28 binds to damaged chromatin in conjunction with HP1, but phosphorylation at S824 reverses its silencing effect. Additionally, Src family kinases phosphorylate TRIM28 near the HP1 box, further reducing its repressive influence by reversing SUMOylation and weakening its association with HP1. These modifications collectively regulate RNAP II pausing during DNA repair and eventually facilitate RNAP II pause release following the completion of DNA repair or transcription-coupled repair [58,59,110,112,113]. TRIM28 has been demonstrated to act as a potent gene repressor when overexpressed in cells. To bind specific DNA sequences, TRIM28 typically associates with Krüppel-associated box (KRAB)-domain-containing zinc finger proteins from the extensive ZNF family. The TRIM28 protein complex often includes the histone methyltransferase SETDB1 and HP1, which facilitate TRIM28 binding to genomic regions enriched in H3K9me3, further underscoring its role in transcriptionally repressive heterochromatin regions of the genome [114,115]. TRIM28 has been shown to regulate HIV transcription both positively and negatively [39,67,68]. Our research, along with that of others, has emphasized TRIM28’s pivotal role in modulating RNAP II activity by regulating its pausing and pause release dynamics [39,58]. Building on this, we discovered that the DNA-PK is crucial in converting TRIM28 from an inhibitor to an activator of HIV transcription via phosphorylation at the S824 residue. Notably, we also found that the cocaine-induced stimulation of HIV transcription operates through this same mechanism. Given TRIM28’s dual function as both a positive and negative regulator of transcription, the direct targeting of TRIM28 may be challenging. However, targeting upstream regulators, like the DNA-PK, presents a promising approach to prevent TRIM28 phosphorylation, potentially suppressing HIV transcription, replication, and latency reactivation.

Our previous findings, where we establish the vital role of the DNA-PK during HIV transcription [37,39], have been extended by others. The role of the DNA-PK in general cell transcription has also been documented [60], validating the important role of the DNA-PK during the basic transcriptional process. To further validate our findings and establish the crucial role of cocaine in stimulating the DNA-PK during HIV transcription, we employed a highly specific human-tested DNA-PK inhibitor, M3814. The dose-dependent inhibition of HIV transcription by M3814, indicated by reduced luciferase gene expression from the LTR promoter (Figure 3B,C), confirmed the direct role of the DNA-PK in promoting HIV transcription. TNF-α was unable to reactivate latent HIV in the presence of M3814, demonstrating that DNA-PK inhibitors hold therapeutic potential to not only effectively suppress HIV transcription and replication but also could be useful in restricting the reactivation of latent HIV provirus (Figure 3E,F). Interestingly, we did not observe any noticeable cell toxicity with the used concentrations of M3814 (Figure 3D), establishing the physiological significance of the findings. Subsequently, we also evaluated the effect of two clinically tested DNA-PK inhibitors, M3814, and NU7441 on HIV replication. We found that more specific DNA-PK inhibitors (DNA-PKi) were better at repressing HIV gene expression and replication (Figure 4). This observation again confirmed the target-specific impact of the DNA-PKi. Moreover, cell viability analysis validated the physiological viability of the pre-clinically and clinically tested DNA-PK inhibitors as potential HIV therapeutics.

Earlier we identified the presence of the DNA-PK at the HIV LTR and its direct role in catalyzing RNAP II CTD phosphorylation [37,39]. In this investigation, we explored whether cocaine-induced HIV transcription and replication result from DNA-PK stimulation and the subsequent phosphorylation of the RNAP II CTD. We specifically examined the state of RNAP II CTD phosphorylation following cocaine exposure. The significant upregulation of Ser2 and Ser5 phosphorylation following cocaine treatment in a dose-dependent manner confirmed the hypothesis that cocaine augments HIV transcription by supporting RNAP II CTD phosphorylation (Figure 6A,B). Ser5 phosphorylation is the marker of transcriptional initiation, and Ser2 phosphorylation is linked to the elongation phase of transcription, including HIV transcription. The data obtained showed that cocaine facilitates both the initiation and elongation phases of transcription. The results were reproduced in cells of multiple lineages to show the ubiquitous prevalence of the observed phenomenon (Figure 6C–F). Subsequently, we explored whether cocaine-enhanced RNAP II phosphorylation is a result of DNA-PK activation using a clinically evaluated, highly specific DNA-PK inhibitor (M3814) in the presence of cocaine (Figure 6E,F). The dose-dependent inhibition of RNAP II CTD phosphorylation at both Ser2 and Ser5 sites by M3814 validated the specific role of the DNA-PK in catalyzing CTD phosphorylation. Altogether, our findings confirmed our hypothesis that cocaine, through the activation of the DNA-PK, significantly influences both the initiation and elongation phases of HIV transcription, contributing to a more comprehensive understanding of the molecular mechanism behind cocaine’s impact on HIV transcription and replication.

Prior studies have established the crucial role of the CDK9 subunit of P-TEFb as a key player in promoting RNAP II processivity by catalyzing phosphorylation of the RNAP II CTD at the Ser2 position, thereby facilitating the elongation phase of transcription [116,117]. In light of this, we aimed to investigate the nuclear levels of P-TEFb. Our analysis indicated that cocaine significantly upregulates CDK9 activity by augmenting its phosphorylation, and in parallel, we also found enhanced levels of cyclinT1, another subunit of P-TEFb. Thus, cocaine further enhances ongoing transcriptional elongation through P-TEFb stimulation. Importantly, cocaine does not affect the total CDK9 levels. Later, we examined the impact of cocaine on the initiation phase of HIV transcription, and, as anticipated, we found the significant upregulation of CDK7, a subunit of TFIIH that is well known to support the initiation phase of transcription, including HIV transcription. These findings were validated in different cell types, both myeloid and lymphoid cells (Figure 7A–F). To further validate that the cocaine-induced phosphorylation of CDK9 and the activation of total CDK7 are indeed reliant on the specific activation of the DNA-PK, we conducted experiments using a DNA-PKcs knockdown cell line exposed to cocaine. In the absence of the DNA-PK, we observed a marked decrease in p-CDK9 (Thr186) levels, as well as a reduction in total CDK9 and CDK7. Notably, in wild-type cells, exposure to cocaine resulted in the anticipated enhancement of CDK7 and CDK9 phosphorylation, consistent with our previous findings. However, in DNA-PKcs knockdown cells, the levels of pCDK9 (Thr186) and CDK7 remained reduced following cocaine exposure, providing strong evidence that cocaine-induced CDK9 phosphorylation and CDK7 activation are specifically mediated by the DNA-PKcs. The impact of cocaine on both the initiation and elongation phases of HIV transcription was further validated by showing the presence of TFIIH (CDK7) and P-TEFb (CDK9), respectively, at the HIV LTR (Figure 10) through ChIP assays upon cocaine treatment. Given that P-TEFb plays a crucial role during the elongation phase, accordingly, we found the enrichment of CDK9 at the downstream region, namely, the Nuc-2 and Env regions of HIV, but highly reduced recruitment at the promoter and Nuc-1 regions (Figure 10). Similarly, after cocaine stimulation, CDK7 was enriched as expected at the LTR promoter but not at the downstream regions, again validating its requirement, especially during the initiation phase of HIV transcription. Our previous findings showed not only the direct interaction between the DNA-PK and RNAP II but also the parallel recruitment of the DNA-PK along with RNAP II at the HIV LTR upon cell stimulation [37,39]. Notably, we confirmed the upregulation of cell metabolism and subsequent cell stimulation following cocaine exposure [27]. These findings prompted us to investigate whether cocaine-mediated cell stimulation and induced DNA-PK activation enhance RNAP II CTD phosphorylation, both via directly catalyzing and through promoting P-TEFb recruitment at the HIV LTR. As expected, we found enhanced CDK9 recruitment at the downstream regions, marking the presence of the elongation complex, along with parallel enrichment profiles of both the DNA-PK and RNAP II across the HIV genome after cocaine treatment. This confirms that cocaine-induced cell stimulation is sufficient not only to activate the DNA-PK (Figure 1) but also to enhance DNA-PK recruitment at the HIV LTR with a proportional increase in RNAP II recruitment at the LTR (Figure 10). Interestingly, the corresponding decrease in the recruitment of H3K27Me3 at the HIV LTR following cocaine treatment demonstrates the loss of a repressive epigenetic structure and establishment of a transcription-supporting euchromatin structure, aligning with our previous findings [39]. Altogether, these findings demonstrate that the cocaine-induced DNA-PK facilitates transcriptional initiation by catalyzing the RNAP II CTD at Ser5. Furthermore, cocaine-mediated DNA-PK stimulation augments the elongation phase of HIV transcription by enhancing the phosphorylation of the RNAP II CTD at Ser2 both via directly catalyzing and promoting the recruitment of P-TEFb.

We also explored whether cocaine could facilitate HIV transcription by promoting RNAP II pause release. We found that cocaine profoundly enhances TRIM28 phosphorylation at its serine 824 residue. This specific phosphorylation event relieves the TRIM28-mediated pausing of RNAP II by converting TRIM28 into a transcription-supporting factor [58,60]. The established interaction between TRIM28 and RNAP II underscores the significant role of TRIM28 in regulating HIV transcription. Additionally, our studies, in line with previous research, have elucidated that the DNA-PK interacts with TRIM28 and catalyzes its phosphorylation at serine 824, resulting in the formation of p-TRIM28-(S824) [39,60]. This phosphorylation event has been associated with positive elongation factors, suggesting its potential role in facilitating the transition from transcriptional pausing to elongation. Consequently, this modification transforms TRIM28 from a transcriptionally repressive factor into a transcriptionally active one. Therefore, we investigated whether cocaine could convert TRIM28 from a transcriptionally repressive factor to a transcriptionally active one by inducing the phosphorylation of TRIM28 at S824. We observed that, upon cocaine exposure, the phosphorylation of TRIM28 at S824 significantly increases in a dose-dependent manner (Figure 8A,B). These findings were confirmed in cells of different lineages, validating the uniformity of the findings (Figure 8C–J). Later, we analyzed both cytosolic and nuclear levels of p-TRIM28 (S824) and TRIM28 upon cocaine exposure. We noted a significant increase in nuclear levels of p-TRIM28 (S824) in cocaine-treated cells, but TRIM28 total did not change significantly (Figure 9A). These findings further validated the cocaine-induced activation and phosphorylation of TRIM28 at S824. Subsequently, we analyzed the recruitment of p-TRIM28-(S824) at the HIV LTR using ChIP assays. As anticipated, we noted enhanced recruitment of phosphorylated TRIM28 (S824) in parallel to DNA-PK recruitment along the HIV genome after cocaine treatment (Figure 10E,F). The accumulation of p-TRIM28 (S824) marks the presence of the transcription-supporting form of TRIM28 and thus indicates the transformation of paused RNAP II into a processive elongating RNAP II. This observation demonstrates that by enhancing the phosphorylation of TRIM28, cocaine effectively alleviates RNAP II pausing, thereby providing essential support to the process of HIV transcription. This is another molecular mechanism through which cocaine influences the regulation of transcriptional processes, specifically within the context of HIV gene expression. We further investigated whether cocaine-induced phosphorylation of TRIM28 at S824 is a result of cocaine-induced DNA-PK activation. Upon treating cells with a highly specific DNA-PK inhibitor, we observed the dose-dependent inhibition of cocaine-induced phosphorylation of TRIM28 at S824 (Figure 8I,J). This finding confirms the critical role played by the DNA-PK in promoting RNAP II pause release by selectively catalyzing TRIM28 phosphorylation at S824 and subsequently promoting HIV transcription following cocaine exposure.

Overall, our findings presented here provide compelling and robust evidence affirming the pivotal role played by cocaine on HIV transcription and gene expression. Our investigations have revealed that cocaine augments HIV transcription by significantly enhancing the nuclear levels of the DNA-PK, besides increasing its catalytic activity through specific phosphorylation at S2056. We found that the cocaine-induced activation of the DNA-PK significantly contributes to various stages of HIV transcription, subsequently bolstering the process of HIV replication. Specifically, the activation of the cocaine-induced DNA-PK assumes a critical role in facilitating transcriptional initiation by augmenting the phosphorylation of the RNAP II CTD at Ser5, alleviating RNAP II pausing through the phosphorylation of TRIM28 at S824 and promoting transcriptional elongation through both the catalysis of CTD phosphorylation at Ser2 and the enhancement of P-TEFb activity. Altogether, our results have distinctly demonstrated that the inhibition or depletion of the DNA-PK results in a strong inhibition of not only cocaine-induced HIV transcription and replication but also HIV transcription induced by other cell stimuli [37,39]. The overall findings suggest a comprehensive insight into the underlying molecular mechanisms by which the cocaine-induced DNA-PK effectively elevates HIV transcription and gene expression (Figure 11).

Additionally, we have demonstrated the translational potential of DNA-PK inhibitors in curtailing HIV gene expression, replication, and the reactivation of latent proviruses [37,39]. These findings advocate for the potential therapeutic application of specific DNA-PK inhibitors as adjuncts in ART regimens, thereby augmenting the efficacy of anti-HIV therapy. It is noteworthy that while ART treatment effectively controls HIV replication, it is ineffective in regulating HIV gene expression from reactivated latent proviruses. These findings strongly advocate for the inclusion of transcriptional inhibitors, such as DNA-PK inhibitors, to supplement ART regimens to mitigate the transient reactivation of latent proviruses, confirmed also by our previous findings involving HIV patients’ samples [39]. DNA-PK inhibitors could also prove beneficial in addressing comorbidities that remain prevalent in well-controlled, ART-adherent PLWH, such as persistent inflammation and immune activation driven by the ongoing presence of HIV proteins. Additionally, DNA-PK inhibitors may help mitigate cocaine-induced toxicities and could potentially lower the incidence of HIV-associated cancers, especially since DNA-PK inhibitors are currently being explored as a treatment for cancer.

The limitation of the study: Our study has a limitation. We did not consistently quantify the precise amount of replication-competent viruses used for cell infection. However, we maintained an equal viral load in both the control (mock) and test samples.

## 5. Conclusions

Understanding the molecular mechanisms governing the HIV life cycle, particularly regarding transcription and replication, is pivotal for developing an HIV cure and achieving eradication. Our research provides compelling evidence highlighting the critical role of the cocaine-induced activation of the DNA-PK in driving key phases of HIV transcription, thereby enhancing viral replication. We found that cocaine significantly upregulates DNA-PK expression, activates the DNA-PK via increased phosphorylation at S2056, and promotes its nuclear localization. This activation facilitates transcriptional initiation by enhancing CTD phosphorylation at Ser5, alleviates RNAP II pausing through TRIM28 phosphorylation at S824, and supports transcriptional elongation by catalyzing CTD phosphorylation at Ser2 and boosting P-TEFb recruitment. Notably, our findings reveal that inhibiting or depleting the DNA-PK significantly impairs cocaine-induced HIV transcription and replication. These results collectively unveil the underlying molecular mechanisms through which the cocaine-induced DNA-PK enhances HIV transcription and gene expression.

## Data Availability

The authors confirm that the data supporting the findings of this study are available within the article or its Supplementary Materials. Further inquiries can be directed to the corresponding author upon reasonable request.

## Supplementary Materials

The following supporting information can be downloaded at https://www.mdpi.com/article/10.3390/cells13231950/s1, Figure S1: Cocaine enhances both the catalytic activity and nuclear level of DNA-PK; Table S1: List of primer sequences.
