# Supplementary material for: Cocaine-Induced DNA-Dependent Protein Kinase Relieves RNAP II Pausing by Promoting TRIM28 Phosphorylation and RNAP II Hyperphosphorylation to Enhance HIV Transcription
Source: Cells. 2024 Nov 23;13(23):1950. doi: 10.3390/cells13231950 (PMC11640508; doi:10.3390/cells13231950)
Supplement: Supplementary file 1 [file cells-13-01950-s001.zip › cells-3306672-supplementary.pdf]

Research Article

# Cocaine-Induced DNA-Dependent Protein Kinase Relieves RNAP II Pausing by Promoting TRIM28 Phosphorylation and RNAP II Hyperphosphorylation to Enhance HIV Transcription

Adhikarimayum Lakhikumar Sharma, Priya Tyagi, Meenata Khumallambam and Mudit Tyagi *

Center for Translational Medicine, Thomas Jefferson University, 1020 Locust Street, Philadelphia, PA 19107, USA;
lakhikumarsharma.adhikarimayum@jefferson.edu (A.L.S.);
ptyagi28@terpmail.umd.edu (P.T.); mxk848@jefferson.edu (M.K.)
* Correspondence: mudit.tyagi@jefferson.edu; Tel.: +1-215-503-5157 or +1-609-509-6709

**Table S1: List of primer sequences.**

| Primer Sets | Primer name | Sequence (5'-3') | Purpose |
|---|---|---|---|
| **1st Set primer** | GAPDHF | CGGGATTGTCTGCCCTAATTAT | Real time PCR |
| | GAPDHR | GCACGGAAGGTCACGATGT | Real time PCR |
| **2nd Set primer** | HIV Promoter F | AGCTTGCTACAAGGGACTTTCC | Real time PCR |
| | HIV promoter R | ACCCAGTACAGGCAAAAAGCAG | Real time PCR |
| **3rd Set primer** | HIV Nuc-1F | CTGGGAGCTCTCTGGCTAACTA | Real time PCR |
| | HIV Nuc-1R | TTACCAGAGTCACACAACAGACG | Real time PCR |
| **4th Set primer** | HIV Nuc-2F | GACTGGTGAGTACGCCAAAA | Real time PCR |
| | HIV Nuc-2R | TTTCCCACTGCGATCTAATTC | Real time PCR |
| **5th Set primer** | HIV envF | TGAGGGACAATCGGAGAAG | Real time PCR |
| | HIV envR | TCTGCACCACTCTTCTCTT | Real time PCR |
| **6th Set primer** | DNA-PKF1 | ACGGTAGGGGAAAGCCATTG | Real time PCR |
| | DNA-PKR1 | CGCTATAGGTCCTCAGCTGC | Real time PCR |
| **7th Set primer** | ActinF1 | AGAGCAAGAGAGGCATCCTG | Real time PCR |
| | ActinR1 | GGGTCATCTTTTCACGGTTGG | Real time PCR |

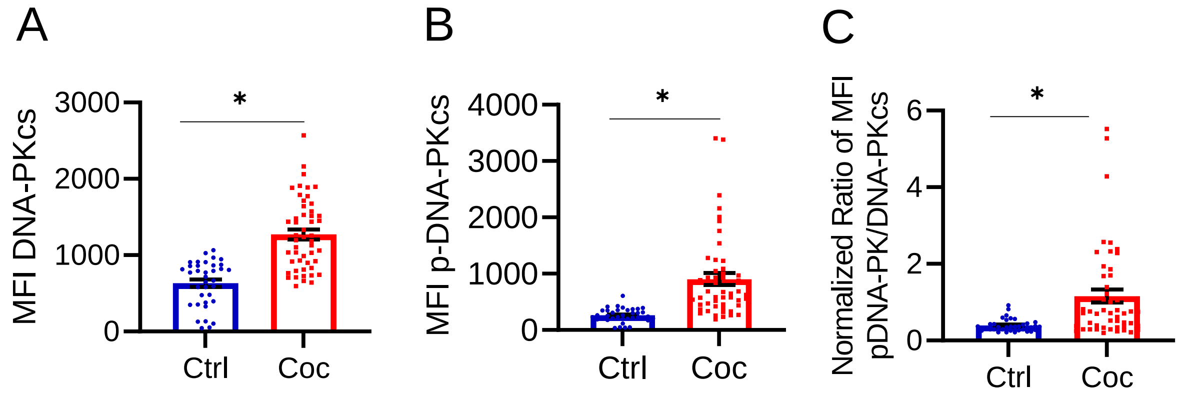


**Supplementary Figure S1: Cocaine enhances both the catalytic activity and nuclear level of DNA-PK. (A, B, & C) Immunofluorescence analysis (mean fluorescence intensity, MFI) of Figure 1, panel I:** Quantitation of the MFI in the number of microglial cells with nuclear p-DNA-PKcs (S2056) and DNA-PKcs (total) proteins in control (Ctrl) Vs. Cocaine (Coc) treated. Bar graph represents the MFI of (**A**) DNA-PKcs, (**B**) pDNA-PKcs (2056) and (**C**) normalized ratio of pDNA-PKcs/DNA-PKcs. Data represent cell numbers counted in five different high-power fields in a representative experiment ± SEM. The results are expressed as mean ± SD and analyzed by one- or two-way ANOVA, followed by Tukey's multiple comparison test. Asterisks over the bars indicate significant differences: $*p < 0.05$ for the comparison of cocaine-treated cells vs. untreated cells (Ctrl).
